# Translation, cross-cultural adaptation, and psychometric validation of the Malay version of the Assessment of Quality of Life—6 Dimensions (Malay-AQoL-6D) instrument among Malaysians living with chronic heart failure

**DOI:** 10.1186/s41687-024-00763-3

**Published:** 2024-07-25

**Authors:** Yi Jing Tan, Siew Chin Ong, Sook Pin Goh, Gang Chen, Vee Sim Yong, Wei Wern Khor, Ying Min Kan, Yong Ying Choong, Ainul Mardhiyyah Zameram, Lin Yuing Tan, James Yau Hon Voo, Kar Kei Lam, Chia How Yen, Mohamed Jahangir Abdul Wahab, Zarina Banu Abdulla

**Affiliations:** 1 Discipline of Social and Administrative Pharmacy, School of Pharmaceutical Sciences, Universiti Sains Malaysia, Gelugor, Penang 11800 Malaysia; 2 Seri Manjung Hospital, Ministry of Health Malaysia, Seri Manjung, Perak 32040 Malaysia; 3 Tapah Hospital, Ministry of Health Malaysia, Tapah, Perak 35000 Malaysia; 4 Centre for Health Economics, Monash University, Caulfield East, VIC 3145 Australia; 5 Clinical Research Centre, Hospital Queen Elizabeth II, Institute for Clinical Research, National Institute of Health, Ministry of Health Malaysia, Kota Kinabalu, Sabah 88300 Malaysia; 6 Sarawak General Hospital, Ministry of Health Malaysia, Kuching, Sarawak 93586 Malaysia; 7 Sungai Dua Health Clinic, Ministry of Health Malaysia, Butterworth, Penang 13800 Malaysia; 8 Tengku Ampuan Afzan Hospital, Ministry of Health Malaysia, Kuantan, Pahang 25100 Malaysia; 9 Teluk Intan Hospital, Ministry of Health Malaysia, Teluk Intan, Perak 36000 Malaysia; 10 Duchess of Kent Hospital, Ministry of Health Malaysia, Sandakan, Sabah 90000 Malaysia; 11 Penang General Hospital, Ministry of Health Malaysia, Georgetown, Penang 10990 Malaysia

**Keywords:** AQoL-6D, EQ-5D-5L, Confirmatory factor analysis, Health-related quality of life, Heart failure, Malay, Psychometric validation, Reliability, Translation and cross-cultural adaptation, Validity

## Abstract

**Background:**

This study aimed to translate and culturally adapt the Assessment of Quality of Life (AQoL)-6D into Malay (Malay-AQoL-6D), and assesses the instrument’s acceptability, reliability, and validity among Malaysians living with chronic heart failure (HF).

**Methods:**

The translation and cross-cultural adaptation process adhered to international guidelines. The Malay-AQoL-6D underwent content and face validity assessments via expert review, and pretesting among healthy individuals and patients with chronic conditions. Subsequent psychometric validation utilised clinico-sociodemographic data and paired AQoL-6D and EQ-5D-5L data from a health-related quality-of-life (HRQoL) survey involving Malay-speaking patients with HF, which encompassed assessments of Malay-AQoL-6D acceptability, internal consistency and test-retest reliability, as well as its construct, concurrent, convergent and divergent, and known-group validity.

**Results:**

The Malay-AQoL-6D was deemed acceptable among clinicians and local patients, achieving a 90.8% completion rate among 314 patients surveyed. The instrument demonstrated strong content validity (item-level content validity index [CVI]: 0.83–1.00, average CVI: 0.98), internal consistency (Cronbach’s alpha: 0.72–0.89; MacDonald’s omega: 0.82–0.90, excluding the Senses dimension), and test-retest reliability (average intraclass correlation coefficients: 0.79–0.95). Confirmatory factor analysis confirmed the instrument’s two-level, six-factor structure (Satorra-Bentler [SB]-scaled χ2(*df*: 164): 283.67, *p*-value < 0.001; root mean square error of approximation [RMSEA]: 0.051; comparative fix index [CFI]: 0.945, Tucker-Lewis index [TLI]: 0.937; standardised root mean-squared error [SRMR]: 0.058). The Malay-AQoL-6D’s concurrent validity was evident through its good agreement with EQ-5D-5L. Multiple hypothesis tests further affirmed its construct and known-group validity. The Malay-AQoL-6D’s psychometric properties remained consistent across different missing data techniques.

**Conclusion:**

The findings suggest that Malay-AQoL-6D could be a culturally acceptable, reliable, and valid HRQoL measure for quantifying HRQoL among the local HF population. Future studies are necessary to further validate the instrument against other measures and confirm the instrument’s test-retest reliability and responsiveness, which are possible with the availability of the Malay-AQoL-6D.

**Supplementary Information:**

The online version contains supplementary material available at 10.1186/s41687-024-00763-3.

## Background

Health-related quality of life (HRQoL) is a multidimensional concept that assesses the extent to which one’s usual or expected physical, mental, and social wellbeing are influenced by their health status, including any medical conditions and related treatments [1]. As a patient-reported outcome measure, HRQoL offers insights into how individuals perceive the impact of the disease and its treatment on their health status from their perspective, rather than that of their clinician. In the case of chronic conditions like heart failure (HF), patients typically contend with troublesome symptoms such as breathing difficulties, fatigue, and fluid retention, coupled with the constant fear of sudden death or hospitalisation due to the sudden worsening of symptoms, all of which having a significant impact on their wellbeing. Previous research consistently demonstrates the prognostic significance of HRQoL assessment in HF management. The advantages include enhanced patient-clinician communication, greater patient participation in their care, increased patient satisfaction, and early detection of unaddressed issues, and improved HRQoL. These improvements translate into reduced adverse outcomes, such as fewer emergency department visits and hospitalisations, and even better survival among HF patients [1–3].

Given its importance, routine assessment of HRQoL among HF patients is currently advocated by international clinical practice guidelines [2]. An extensive selection of instruments is available for assessing HRQoL among the HF population, encompassing both generic and disease-specific options. While generic measures may be less sensitive to clinical changes, they offer the advantage of detecting the impact of treatment-associated complications or other comorbidities that might otherwise be overlooked when relying solely on disease-specific measures [3]. Furthermore, generic measures can be used to derive population norms [4, 5] and enable comparisons across different medical conditions. Preference-based generic instruments, such as the EQ-5D-5L [6] and the Assessment of Quality of Life (AQoL)-6D [7], can further be used to generate health state utility values (HSUVs), which are necessary to support economic evaluations. These economic analyses are necessary to inform decisions regarding healthcare resource allocation. The current consensus is that both generic and HF-specific questionnaires should be co-administered where possible, although this may lead to increased respondent burden [3].

The AQoL-6D, formerly known as AQoL II, was first introduced by Monash University in 2003. The instrument comprises 20 items organised into six health dimensions: Independent Living, Relationships, Mental Health, Coping, Pain, and Senses. The AQoL-6D was developed based on the concept of *handicap*, which relates to the impact of an individual’s health status on their functioning within their *social context*, rather than merely whether they can or cannot carry out certain functions [8]. Widely used in Australia, the AQoL-6D caters to both general population and diverse patient groups, with proven validity, reliability, and responsiveness [9, 10]. Within the Malaysian healthcare and research context, the AQoL-6D represents an appealing alternative to the more commonly used EQ-5D instrument [11] for assessing HRQoL among HF patients. Besides being accessible in multiple languages at no cost [12], the AQoL-6D boasts an expanded scope, less pronounced ceiling effect, and enhanced sensitivity in capturing subtle differences in HRQoL, particularly among individuals who may experience psychosocial issues while remaining physically well. In comparison to the EQ-5D, although some conceptual overlap exists [13], items within the Relationships, Coping, and Senses dimensions are unique to the AQoL-6D and can better address problems experienced by HF patients.

In Malaysia, routine HRQoL assessment is not yet implemented in HF clinics. In the effort to explore the feasibility and promote such implementation, a nationwide, cross-sectional HRQoL survey known as the HRQoL-HF-MOH study was conducted. The survey applied both the EQ-5D-5L and AQoL-6D instruments to gain insights into HRQoL among the local HF outpatients as well as to investigate the suitability of the AQoL-6D for this purpose. The AQoL-6D is already available in English and Chinese, which can be understood by within the multilingual Malaysian community. However, to ensure even broader accessibility among the local multi-ethnic populace, translation of the AQoL-6D into the national unifying language, Bahasa Malaysia (or Malay), is warranted. Having a common language version of the instrument, understood by all Malaysians, for HRQoL assessment is convenient for both patients and clinicians and will allow inter-racial group comparisons.

To this end, the current study has three objectives: (1) to translate the AQoL-6D from English to its Malay version (referred to as the Malay-AQoL-6D); (2) to conduct content and cross-cultural validation of the Malay-AQoL-6D among healthy adults and those with chronic diseases, including HF; and (3) to psychometrically validate the Malay-AQoL-6D for HRQoL assessment within the local HF population, including assessments of its acceptability, construct, convergent and discriminant, concurrent and know-group validity, as well as internal consistency and test-retest reliability, using the HRQoL-HF-MOH data.

## Methods

### Translation, cross-cultural adaptation, and content validation

**Fig. 1:**
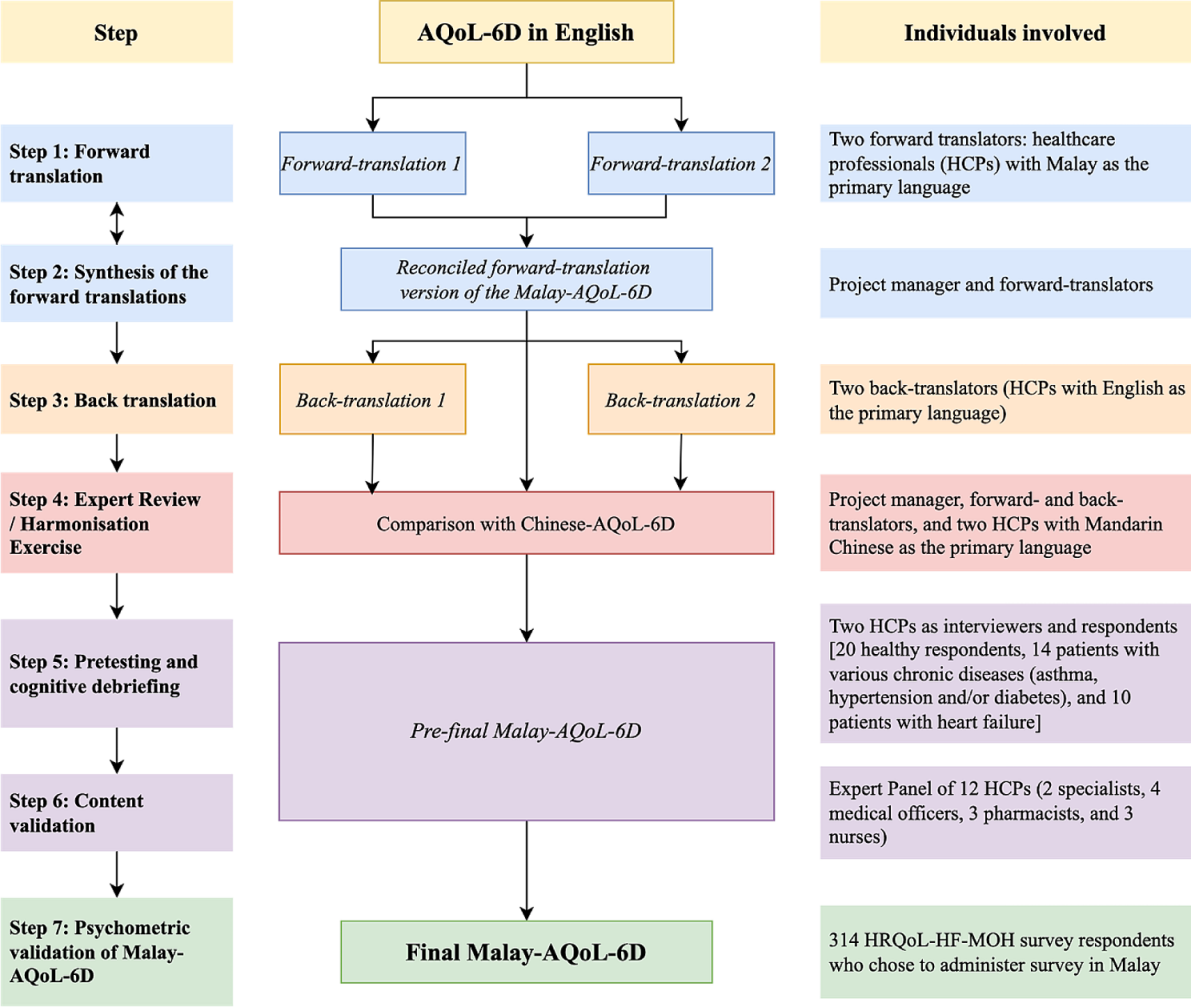
The process of translation, cross-cultural adaptation, and validation

The translation and cross-cultural adaptation of the AQoL-6D into Malay was conducted in compliance with guidelines by Beaton et al. [14], the World Health Organisation [15], and the Professional Society for Health Economics and Outcomes Research (ISPOR) [16–20]. Permission to translate, culturally adapt, and validate the AQoL-6D in Malay was obtained from the original developer whose guidance and source documents were pivotal in ensuring accurate interpretation of items or concepts. All individuals involved in the translation and cross-cultural adaptation process were healthcare professionals (HCPs) in the country with bilingual or trilingual capabilities (Malay, English, and/or Mandarin Chinese). Figure 1 illustrates the work process involved in the translation, cross-cultural adaptation, pretesting, and psychometric validation of the Malay-AQoL-6D. After briefing by the project manager to establish understanding about the concepts and items underlying the instrument, two Malay-speaking HCPs independently forward-translated the original English version into Malay. The project manager then merged the forward-translations into a reconciled version, resolving any discrepancies through discussions. Subsequently, two HCPs, proficient English users and unaware of the original English version, independently back-translated the reconciled Malay version into English. The project manager compared these back-translations with the original to identify discrepancies and omissions. Lastly, a harmonisation exercise, involving the project manager, both forward- and back-translators, and two additional Mandarin-speaking HCPs, compared the Chinese version with the English and Malay versions to further refine pre-final Malay version for ensuring equivalence among the three language versions.

An expert panel of 12 HCPs, including two specialists, four medical officers, three pharmacists, and three nurses), was convened to evaluate the content validity of the pre-final Malay-AQoL-6D. The assessment was made against the 11 criteria stated in the modified version of the Simon and White’s Survey Validation Rubric for Expert Panel [21] (refer to Online Supplementary Section 3, S3.1 for further details). Content validity indices (CVI), including item-level CVI (I-CVI) and average-CVI (Ave-CVI) were then calculated. A content-validated instrument would yield I-CVI and Ave-CVI values of at least 0.78 and 0.90, respectively [22].

Next, pre-testing and cognitive debriefing exercise were conducted with three distinct target populations, including 20 healthy respondents, 10 patients with chronic heart failure, and 14 patients with other chronic diseases, including asthma, hypertension, and/or diabetes. The pre-final Malay-AQoL-6D was initially administered to the respondents, who were then subject to a cognitive debriefing conducted by two experienced interviewers. During the debriefing, respondents were asked to interpret each question in their own words and explain their chosen answer. Verbal responses were compared with their AQoL-6D responses for consistency. At the end of each session, respondents were invited to share if they found any word or expression used difficult to understand, unacceptable or offensive.

### Psychometric validation

#### Source of validation sample: the HRQoL-HF-MOH study

The design and reporting of psychometric validation of the Malay-AQoL-6D were guided by the Consensus-based Standards for the selection of health Measurement Instruments (COSMIN) checklists. Moreover, the current study referenced the definitions of psychometric properties and performance criteria compiled by Kwon et al.(2023) [30], which incorporated insights from multiple established standards and guidelines. These included the COSMIN checklists, as well as guidelines by the International Society for Quality of Life Research, the U.S. Food and Drug Administration, and the Medical Outcomes Trust.

The data for psychometric validation of the Malay-AQoL-6D among HF patients were sourced from the HRQoL-HF-MOH survey, as mentioned above. The survey, registered with the National Medical Research Register of Malaysia (NMRR ID-23-00340-PWE) and approved by the Medical Research and Ethics Committee, was conducted from April to September 2023, targeting HF outpatients treated in public hospitals managed by the Ministry of Health across the country. A multi-stage, purposive sampling approach was adopted to ensure a diverse sample of HF patients from different locations and sociodemographic backgrounds. Eligible participants included adults aged 18 years or older diagnosed with HF for at least three months who could understand either Malay, English or Mandarin Chinese. Caregiver- (proxy-) administered survey was allowed for patients unable to provide responses. Excluded were hospitalised patients, those with documented cognitive impairment, or any condition hindering survey completion. Furthermore, individuals with active cancer, recent radio- or chemotherapy, end-stage non-cardiac diseases, or those under palliative/hospice care for non-HF related conditions were ineligible to participate in this study. Patients and their proxies were invited to participate during their clinic visits and requested to complete a survey form in their preferred language (Malay, English, and Mandarin) upon providing written consent. The survey form collected the patient’s sociodemographic details alongside EQ-5D-5L and AQoL-6D responses. Respondents or proxies were encouraged to fill out the survey form on their own, with assistance available from the data collector if needed. Clinical data were collected separately from medical records.

The EQ-5D-5L is made up of a descriptive system and a visual analogue scale (EQ-VAS), where respondents describe their health state on the same day they complete the questionnaire. The EQ-VAS requires respondents to rate their own health from 0 (the worst imaginable health) to 100 (the best imaginable health). The descriptive system has 5 questions representing 5 health dimensions in the following order: Mobility, Self-care, Usual Activities, Pain/Discomfort, and Anxiety/Depression. Each question offers 5 response levels (1 to 5) to indicate varying degrees of problem severity, ranging from no problem (level 1) to extreme problem (level 5). Responses to all questions can be summarised, in the sequence of the five domains, as a 5-digit health profile (e.g., 12312), which can be further converted into a HSUV using the Malaysian value set [23]. The psychometric properties of the EQ-5D-5L instrument have been validated among general population [24, 25] and across different disease areas including heart diseases [26, 27].

The AQoL-6D comprises 20 items grouped into 6 dimensions, encompassing Independent Living, Relationships, Mental Health, Coping, Pain, and Senses [7]. There are 3 to 4 items within each health dimension and 4 to 6 response levels per item, where a higher level indicates a more severe problem. The recall period is within the past 7 days. As a psychometric measure, the response scores for all items within each dimension are summed and transformed onto a 0 to 100 scale using an unweighted scoring algorithm [28]. A higher score indicates a better HRQoL. The scores for all six dimensions are generated separately, alongside a global score. Individual HSUVs can also be calculated based on the utility weights, which were derived from a sample of Australian adult general population using the time trade-off technique [8]. Currently, there is no Malaysian value set available. Online Supplementary Section 1, S1.1 summarises the overlap in health dimensions and differences between the AQoL-6D and the EQ-5D-5L.

#### Sample size determination

A review by White (2022), including 1750 instrument validation studies, recommends a sample size between 250 and 350 patients [29]. Moreover, the widely accepted rule of thumb for sample size for confirmatory factor analysis (CFA) suggests having 5–10 participants per item. Therefore, a sample of 280 subjects, factoring in a 10% incomplete responses rate, was deemed adequate for the validation of Malay-AQoL-6D. In the HRQoL-HF-MOH study, 424 individuals with HF from seven public hospitals across Malaysia participated, achieving a response rate of 85.8% (refer to Online Supplementary Section 4 for recruitment details). Among them, 314 completed the survey in Malay, with 285 (90.7%) completing all Malay-AQoL-6D items. For psychometric validation of Malay-AQoL-6D, only complete cases were used.

#### Descriptive statistics and acceptability of the Malay-AQoL-6D

Descriptive statistics were used to summarise the sociodemographic and clinical characteristics of respondents, as well as their HRQoL data. The acceptability of the Malay-AQoL-6D was determined based on an analysis of missing data, floor and ceiling effects, and score distribution. To be considered as acceptable, the instrument needed to meet at least two of the following criteria: missing data < 5%, floor and ceiling effects < 10%, and > 0.4 unique health states > 0.4 per respondent [30]. Moreover, the proportions of self- and proxy-reports, along with the number of respondents who required assistance from a study team member to complete the survey, were determined.

#### Reliability

Internal consistency for each AQoL-6D dimension was assessed using Cronbach’s alpha, item-rest correlation, and MacDonald’s omega coefficients, and values ≥ 0.7, ≥ 0.2, and ≥ 0.7 were considered satisfactory, respectively [30, 31]. Test-retest reliability was evaluated by having respondents who self-completed using the Malay-AQoL-6D readminister the instrument within 7–14 days through an electronic form delivered via WhatsApp messages. Absolute-agreement intraclass correlation coefficients (ICCs) for two-way mixed models were determined, with values < 0.5, 0.5-<0.7, 0.7-<0.9, and ≥ 0.9 indicating poor, moderate, good, and excellent test-retest reliability, respectively [32].

#### Construct validity

A CFA was conducted using the structural equation modelling approach to validate the established two-level, six-factor structure of AQoL-6D [7–9]. The maximum likelihood estimation, with or without Satorra-Bentler (SB) correction based on data normality, was employed. Items with factor-loading coefficients ≥ 0.4 were deemed sufficiently representative of the underlying factor [30]. Multiple goodness-of-fit indices, including Chi-square (χ^2^) and associated degrees of freedom (*df*) and *p*-value; root-mean-square error of approximation (RMSEA) along with its 90% confidence intervals (CI) and close fit *p*-value (pclose); confirmatory fix index (CFI), Tucker-Lewis index (TLI); and standardised root-mean-square residual (SRMR), were used to evaluate the measurement model. SB-scaled statistics were also reported if SB correction was applied. A good model fit was indicated by the following cut-off values: χ^2^*p*-value > 0.05; RMSEA and its upper 90% CI ≤ 0.08, pclose > 0.05; TLI and CFI ≥ 0.95; SRMR ≤ 0.08 [33].

#### Concurrent validity

Due to extensive evidence supporting EQ-5D-5L validity, the Malay-AQoL-6D metrics were compared with EQ-5D-5L to confirm the concurrent validity of the AQoL-6D. The levels of agreement between the HSUVs from both instruments and between EQ-VAS and AQoL-6D global score were assessed using ICCs. The strength of agreement was based on the same thresholds above. Bland-Altman plots were used to visualise agreement between the two instruments by plotting the differences between the HSUVs (EQ-VAS/AQoL-6D global score) against their mean, along with the 95% limits of agreement (LOA).

#### Convergent and discriminant validity

Convergent and discriminant validity was assessed by testing a set of a priori hypotheses using either the Pearson’s or Spearman rank correlation based on data normality. It was hypothesised a priori that similar health dimensions from both instruments would elicit responses which demonstrate at least moderate correlation (correlation coefficient, *r* ≥ 0.4), while dissimilar health domains would display weak correlation (*r* < 0.4). The hypothesised pairwise correlations among health domains of the two instruments were derived from the literature [7] (Table 3, upper row). Negative correlations were expected between EQ-5D-5L average responses and the corresponding AQoL-6D domain scores, as higher EQ-5D responses indicate more severe problems, while higher AQoL-6D domain scores indicate less severe problems. Conversely, positive correlations were expected between EQ-VAS and AQoL-6D scores, as higher scores indicate more problems across both metrics. Convergent and discriminant validity was confirmed if the results of hypothesis testing were consistent with most a priori hypotheses.

#### Known-group validity

Known-group validity of the Malay-AQoL-6D was established by examining the extent to which its scores (either dimension score, global score, or both) could differentiate between groups with expected differences in HRQoL, guided by prior research. In their systematic review of 29 studies exploring predictors of HRQoL among HF outpatients [34], Baert and colleagues demonstrated significant associations between various patient characteristics with the sum scores of HRQoL measured using disease-specific instruments. Building on these findings, seven hypotheses were formulated a priori to examine the differences in AQoL-6D global score between patient subgroups: (1) Younger patients would report a lower average AQoL-6D global score than older patients. (2) Female patients would report lower AQoL-6D global scores than male patients. (3) Patients with more functional limitation (New York Heart Association [NYHA] functional class III-IV) would report worse AQoL-6D global scores than those with less functional impairment (NYHA class I-II). (4) Patients with more comorbidities (i.e., having a higher Charlson Comorbidity Index [CCI]) would report lower AQoL-6D global scores. (5) Patients with comorbid diabetes mellitus (DM) would report worse AQoL-6D global scores than non-diabetic patients. (6) Patients who are anaemic would report lower AQoL-6D global scores than those without anaemia. (7) Patients requiring high-dose diuretic therapy would report lower AQoL-6D global scores than those who do not. Notably, individual AQoL-6D dimension scores were also compared between patient groups to explore which specific dimensions exhibit significant differences.

Depending on data normality, between-group differences in HRQoL scores were assessed for statistical significance using independent t-tests (one-way ANOVA) or Mann-Whitney U (Kruskal-Wallis H) tests. Known-group validity was confirmed if hypothesis testing yielded statistically significant results consistent with predefined hypotheses. Mean differences between two groups were quantified using Cohen’s *d*, as a measure of effect size, with values of 0.2-<0.5, 0.5-<0.8, and ≥ 0.8 indicating small, medium, and large effects, respectively. For variables with three or more groups, post-hoc (Dunn’s) tests were conducted to identify which two groups were significantly distinct. Bonferroni-corrected *p*-values were reported.

#### Missing data techniques and robustness of validation results

Upon confirming whether the missing responses satisfied the assumption of missing completely at random (MCAR) [35], common techniques such as robust full information maximum likelihood (RFIML), multiple multivariate normal imputation (MI-MVN) [36], and mean imputation were used to address missing values. Of note, mean imputation, built into the AQoL-6D utility scoring algorithm [37], was used to replace missing responses with the rounded average responses from the other items within the same dimensions. However, this method was not applicable for dimensions with ≥ 2 item non-responses. The validation results resulting from different analytical methods: (a) complete-case analysis; (b) RFIML; (c) mean imputation; and (d) MI-MVN, were compared to establish the robustness of psychometric properties.

#### Statistical analyses and software

The utility scoring algorithm available on the official website was used to convert AQoL-6D item responses into utility values [37]. Data normality was assessed through visual inspection (histogram and normal-quantile plots) and the Shapiro-Wilk test. All statistical analyses, including MI, were conducted using Stata version 14.2. Additional user-written Stata modules used included *omegacoef* for determining MacDonald’s omega coefficients [48], *dunntest* for Dunn’s test [38], *mdesc* [39] and *missings* [40] for missing value analysis; *mcartest* for Little’s MCAR test [41], and *blandaltman* for Bland-Altman analysis [42]. Two-sided *p*-values < 0.05 were considered statistically significant.

## Results

### Cross-cultural and content validity of the Malay-AQoL-6D

The AQoL-6D was translated to Malay and back-translated to English as planned. No items were excluded during the cross-cultural adaptation process to ensure the instrument’s measurement equivalence across languages. While there were some adjustments made to address readability and understandability issues, there were no major discrepancies nor omissions in both forward- and back-translations. The harmonisation exercise confirmed the equivalence between the Malay and Chinese versions. During pre-testing, all 44 respondents provided consistent verbal and instrumental responses, and generally considered the language used to be easily comprehensible, acceptable, and non-offensive. The items that required further discussion and revision of the translation are listed in Online Supplementary Section 2. The final Malay-AQoL-6D version, alongside the original English AQoL-6D are included in Online Supplementary Section 1, S1.2 and S1.3.

Expert evaluations of the Malay-AQoL-6D yielded I-CVI scores ranging from 0.83 to 1.00, with an Ave-CVI of 0.98, indicating strong content validity. Further details on content validity index calculations are available in Online Supplementary Section 3, S3.2.

### Characteristics of study participants

The HF cohort surveyed in Malay (*n* = 285) had a mean age of 55.2 years, comprising 24.2% women, 53.3% of Malay descent, and 46.7% of other ethnicities. The majority had at least secondary school education (72.1%), a reduced left ventricular ejection fraction (68.7%), and mild symptoms (90.9% classified as NYHA class I-II). The mean Charlson Comorbidity Index was 4, with 41.8% having comorbid DM. Approximately 75% were receiving at least three agents of the guideline-directed medical therapy [2]. Table 1 summarises the characteristics of the Malay-speaking HF cohort.

**Table 1:** Characteristics of respondents who completed the survey in Malay*

Characteristic	*N* = 285
Age, years	55.2 ± 13.2
Female sex—no. (%)	69 (24.2)
Race—no. (%)	
Malay	152 (53.3)
Chinese	8 (2.8)
Indian	25 (8.8)
Sarawak Dayak	16 (5.6)
Kadazan-Dusun (Sabah native)	46 (16.1)
Bajau (Sabah native)	12 (4.2)
Bugis/Jawa (Sabah native)	9 (3.6)
Bruneian Malay (Sabah native)	10 (2.5)
Other or unspecified^#^	7 (2.5)
Highest education level—no. (%)	
No formal education	23 (8.1)
Primary education	56 (19.7)
Secondary education	162 (57.0)
Degree or diploma	43 (15.1)
Source of survey—no. (%)	
Self-completed	205 (71.9)
Caregiver-proxy completed	80 (28.1)
Assistance from study team when filling out survey form—no. (%)	
Yes	59 (20.7)
No	226 (79.3)
NYHA functional class—no. (%)	
I	149 (52.7)
II	108 (38.2)
III	25 (8.8)
IV	1 (0.4)
LVEF subgroups—no. (%)	
HFrEF: LVEF ≤ 40%	195 (68.7)
HFmrEF: LVEF 41–49%	50 (17.6)
HFpEF: LVEF ≥ 50%	39 (13.7)
Charlson comorbidity index subgroup—no. (%)	
1–4	177 (62.1)
≥ 5	108 (37.9)
Comorbidity—no. (%)	
Anaemia^§^	34 (11.9)
Atrial fibrillation	41 (14.4)
Chronic kidney disease (eGFR < 60 ml/min/1.73 m^2^)	68 (23.9)
Diabetes mellitus	119 (41.8)
History of acute coronary syndrome	140 (50.9)
Number of guideline-directed medical therapy^¶^—no. (%)	
Monotherapy or none	28 (10.5)
Dual therapy	40 (14.1)
Triple therapy	121 (42.5)
Quadruple therapy	94 (33.0)
High-dose diuretic^‡^	17 (6.0)

*eGFR* estimated glomerular filtration rate, *Hb* serum haemoglobin level, HF heart failure, *HFrEF* HF with reduced ejection fraction, *HFmrEF* heart failure with mildly reduced ejection fraction, *HFpEF* heart failure with preserved ejection fraction, *LVEF* left ventricular ejection fraction, *NYHA* New York Heart Association

*Plus-minus values are means ± SD unless mentioned otherwise

^#^Other ethnicities mainly comprised Sabah ethnicities, including Kedayan (*n* = 1), Kayan (*n* = 1), Sungai (*n* = 1), Suluk (*n* = 1), and Murut (*n* = 1); and unspecified (*n* = 2)

^§^Defined as haemoglobin level < 12 g/dL in women or < 13 g/dL in men

^‡^Defined as frusemide equivalent dose ≥ 80 mg per day or combination of two non-MRA diuretics (e.g., a loop diuretic + a thiazide-like diuretic)

^¶^May comprise one or more drugs from the four pillars of guideline-directed medical therapy, encompassing angiotensin-converting enzyme inhibitor, angiotensin receptor blocker, or angiotensin receptor/neprilysin inhibitor, beta-blocker, mineralocorticoid antagonist receptor, and sodium-glucose cotransporter-2 inhibitor

### Distribution of HRQoL scores

Table 2 presents the descriptive statistics of the HRQoL scores derived from the Malay versions of both EQ-5D-5L and AQoL-6D. The mean (median) HSUVs for EQ-5D-5L and AQoL-6D were 0.837 (0.880) and 0.763 (0.809), respectively. The mean (median) EQ-VAS score and AQoL-6D global score stood at 79 (80) and 76 (77), respectively. All scores exhibited a left-skewed distribution, as illustrated in Online Supplementary Section 4, S4.3.

**Table 2:** Distribution of HRQoL scores

HRQoL measure	Theoretical range	Observed range	Mean (SD)	Median (25th and 75th percentile)	Ceiling effect—no. (%)	Floor effect—no. (%)
**EQ-5D-5L**						
HSUV*	−0.442 to 1.000	0.033 to 1.000	0.837 (0.186)	0.880 (0.734, 1.000)	97 (34.0)	0 (0.0)
EQ-VAS	0 to 100	5 to 100	79 (17)	80 (70, 90)	28 (9.8)	0 (0.0)
**AQoL-6D**						
HSUV^#^	−0.044 to 1.000	0.151 to 1.000	0.763 (0.196)	0.809 (0.632, 0.929)	21 (7.4)	0 (0.0)
Global score	0 to 100	34 to 100	76 (14)	77 (66, 86)	2 (0.7)	0 (0.0)
Dimension score						
Independent living	0 to 100	11 to 100	72 (22)	78 (61, 89)	38 (13.3)	0 (0.0)
Relationships	0 to 100	0 to 100	79 (21)	80 (70, 90)	68 (23.9)	2 (0.7)
Mental health	0 to 100	13 to 100	76 (19)	75 (63, 94)	49 (17.2)	0 (0.0)
Coping	0 to 100	0 to 100	66 (22)	73 (54, 82)	19 (6.7)	2 (0.7)
Pain	0 to 100	10 to 100	77 (19)	80 (70, 100)	65 (22.8)	0 (0.0)
Senses	0 to 100	39 to 100	83 (11)	85 (77, 92)	33 (11.6)	0 (0.0)

*AQoL-6D* the Assessment of Quality of Life—6 Dimensions instrument, *EQ-5D-5L* the five-level version of the EuroQoL 5 dimensions instrument, *EQ-VAS* EuroQol visual analogue scale, *HRQoL* health-related quality of life, *HSUV* health state utility, *SD* standard deviation

*Based on Malaysian valuation set for EQ-5D-5L [23]

^#^Based on Australian valuation set for AQoL-6D [8]

### Psychometric properties of the Malay-AQoL-6D

#### Acceptability

All 314 Malay survey respondents completed EQ-5D-5L, with only one not responding to EQ-VAS. The overall nonresponse rate of the Malay-AQoL-6D in this cohort was low at 0.5% (34/6280 patient-questions). Specifically, 25 respondents (8.0%) missed just one item, 3 (1.0%) missed two questions from different health dimensions, and 1 (0.3%) missed three questions, all within the Relationships dimension. Response rates for the six dimensions were nearly perfect, approaching 100%, except for the Relationships dimension, which had a response rate of 91.1%. The items with the highest non-response rates were item-5 (*intimacy*) at 7.3%, and item-7 (*social function*) at 1.3%, both falling under the Relationships dimension (see Online Supplementary Section 7, S7.1 for more details).

In the complete-case dataset (*n* = 285), the Malay-AQoL-6D exhibited low ceiling effects, with only 7.4% reaching the maximum HSUVs and 0.7% for global scores, without observable flooring effects (Table 2). There were 202 unique health states, averaging 0.71 per respondent. These metrics provide compelling evidence of the Malay-AQoL-6D’s acceptability among local HF patients. Of note, the majority (71.9%) of the surveys were completed by the patients themselves, with the remaining 28.1% reported by caretaker-proxies. Most surveys were completed independently, while 20.7% of surveys, whether self- or proxy-reported, required some form of assistance from the study team.

#### Reliability

The Malay-AQoL-6D demonstrated strong internal consistency for five of the six domains, with the exception of the Senses dimension. Cronbach’s alpha and MacDonald’ omega values ranged from 0.72 to 0.89 and 0.82 to 0.90, respectively, across all other health dimensions—except for the Senses dimension, where values were 0.36 and 0.44, respectively. While most items had item-rest correlations exceeding 0.5, the three items in the Senses dimension had coefficients around 0.2.

Test-retest reliability, assessed through ICCs derived from baseline and repeat Malay-AQoL-6D data, ranged from 0.79 to 0.93 (*p* < 0.001) across all dimensions, indicating good to excellent test-retest reliability. However, it is worth noting that the ICCs had wide confidence intervals due to small sample size (*n* = 15). Detailed results are available in Online Supplementary Section 5.

#### Construct validity

**Fig. 2:**
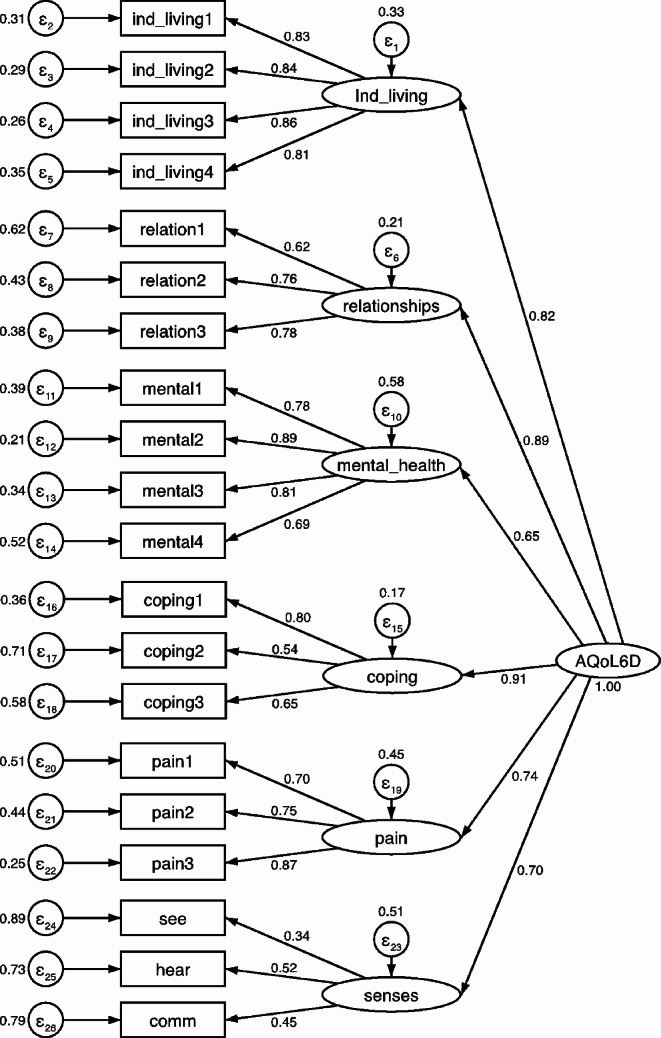
Two-level, six-factor structural model of the Malay-AQoL-6D. *Note* The AQoL-6D consists of 20 items organised in the order as above: 4 items [items no. 1 to 4] under the Independent Living dimension, denoted as ind_living1 to ind_living4; 3 items [items no. 5 to 7] under the Relationship dimension, denoted as relation1 to relation3; 4 items [items 8 to 11] under the Mental Health dimension, denoted as mental1 to mental4; 3 items [items 12 to 14] under the Coping dimension, denoted as coping1 to coping3; 3 items [items 15 to 17] under the Pain Dimension, denoted as pain1 to pain3; and 3 more items [items 18 to 20] under the Senses dimension, denoted by see, hear and comm. Standardised factor-loadings were reported, all having *p*-value < 0.001. ϵ denotes the error terms. From the left, the four sets of numbers represent the error terms on each item, the loadings between the items and their respective dimensions, the error terms on each dimension, and the factor loadings between latent variables and the AQoL-6D

The results of the CFA (Fig. 2), derived from data collected using the Malay-AQoL-6D, confirmed the previously established two-level, six-factor structure of AQoL-6D. Maximum likelihood estimation with Satorra-Bentler correction (ML-SB) was employed due to the non-normal distribution of HRQoL scores. Item loadings on their respective dimensions were generally above 0.4, except for item-19 (*seeing*), which had a loading of 0.34 for the Senses dimension. The factor loadings between the six latent dimensions and the overarching HRQoL construct were all above 0.6. Goodness-of-fit indices suggested an acceptable fit of the model to the data [χ^2^ (*df = 164*) = 352.86, *p*-value < 0.001; SB-scaled χ^2^ (*df = 164*) = 283.67, *p*-value < 0.001; RMSEA = 0.064 (90% CI 0.054, 0.073); RMSEA-SB = 0.051; CFI = 0.932, TLI = 0.921, CFI-SB = 0.945, TLI-SB = 0.937; SRMR = 0.058]. Additional details of the CFA can be found in Online Supplementary Section 6, S6.1.

#### Concurrent validity

**Fig. 3:**
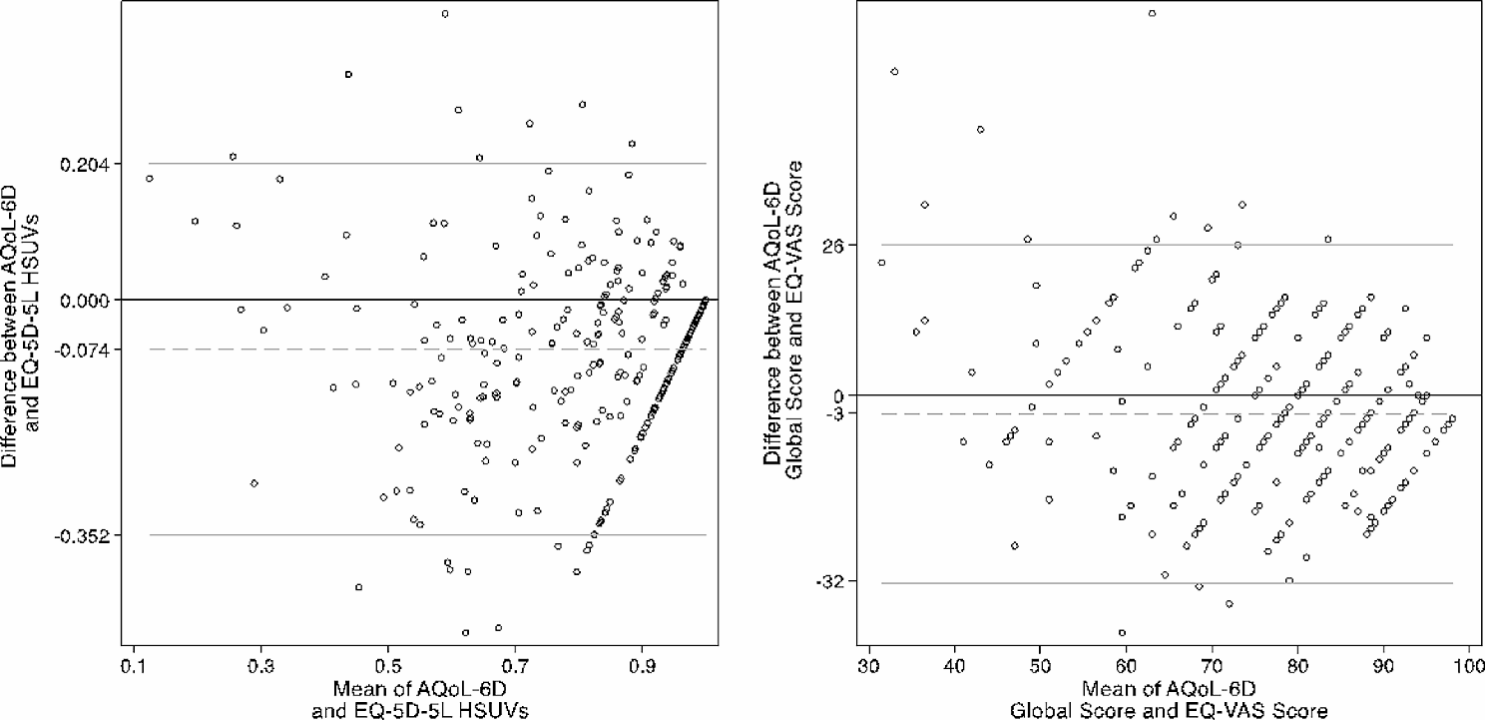
Bland-Altman plots. The grey, dotted line represents the mean difference between the two instruments, while the grey, solid lines represent the 95% limits of agreement. *AQoL-6D* the Assessment of Quality of Life—6 Dimensions instrument, *EQ-5D-5L* the five-level version of the EuroQoL 5 dimensions instrument, *EQ-VAS* EuroQol visual analogue scale, *HSUVs* health state utility values

The HSUVs obtained from EQ-5D-5L and AQoL-6D exhibited a strong level of agreement, as evidenced by an average ICC of 0.81 (95% CI 0.65–0.88, *p* < 0.001). Additionally, the EQ-VAS score exhibited good agreement with the AQoL-6D global score, with an average ICC = 0.70 (95% CI 0.62, 0.76, *p* < 0.001). Bland-Altman plots in Fig. 3 illustrate the agreement between the two instruments in terms of HSUVs and sum scores. The mean difference between the two sets of HSUVs was − 0.074, while the mean difference between the EQ-VAS and the AQoL-6D global score was − 3. The 95% LOA were between − 0.352 and 0.204 for HSUVs, and between − 32 and 26 for the comparison between the EQ-VAS and AQoL-6D global score.

#### Convergent and discriminant validity

Spearman’s rank correlation coefficients were used to examine 42 a priori hypotheses regarding the pairwise correlations among scores obtained using both instruments. The hypotheses were formulated based on the conceptual overlap of the two instruments, as postulated by the AQoL-6D developer [13] (also see Online Supplementary Section 1, S1.1). The hypothesised (upper-row) and actual (lower-row) correlations are presented in Table 3. Out of the 14 hypotheses tested for evaluating discriminant validity (i.e., weak correlations between dissimilar health dimensions), 12 yielded the anticipated results. Moreover, among the 28 predefined hypotheses for assessing convergent validity (i.e., anticipating at least moderate correlations between similar health dimensions), 23 yielded the expected correlations. These findings strongly support the convergent and discriminant validity of the Malay-AQoL-6D.

**Table 3:** Hypothesised and actual correlation coefficients among the EQ-5D-5L and AQoL-6D dimension scores, EQ-VAS, and AQoL-6D global score

		AQoL-6D dimension and global scores (*higher score*,* better HRQoL)*						
		Independent living	Relationships	Mental health	Coping	Pain	Senses	Global score
EQ-5D average item response (*higher score*,* worse HRQoL)*	Mobility	*Moderate-strong*	*Moderate-strong*	*Weak*	*Moderate-strong*	*Moderate-strong*	*Weak*	*Moderate-strong*
		**−0.61**	**−0.49**	**−0.35**	**−0.54**	**−0.39**	**−0.31**	**−0.64**
	Self-care	*Moderate-strong*	*Moderate-strong*	*Weak*	*Moderate-strong*	*Weak*	*Weak*	*Moderate-strong*
		**−0.40**	−0.31	−0.18*	**−**0.31	**−0.22**	**−0.22** ^**#**^	**−**0.38
	Usual activities	*Moderate-strong*	*Moderate-strong*	*Weak*	*Moderate-strong*	*Moderate-strong*	*Weak*	*Moderate-strong*
		**−0.64**	**−0.55**	**−0.34**	**−0.55**	**−0.47**	**−0.29**	**−0.66**
	Pain or discomfort	*Moderate-strong*	*Weak*	*Moderate-strong*	*Moderate-strong*	*Moderate-strong*	*Weak*	*Moderate-strong*
		**−0.51**	**−**0.40	**−0.39**	**−0.45**	**−0.58**	**−0.20** ^**#**^	**−0.58**
	Anxiety or depression	*Weak*	*Weak*	*Moderate-strong*	*Moderate-strong*	*Weak*	*Weak*	*Moderate-strong*
		**−0.37**	**−0.33**	**−0.43**	**−**0.30	**−0.35**	−0.17*	**−0.44**
EQ-VAS (*higher score*,* better HRQoL*)		*Moderate-strong*	*Moderate-strong*	*Moderate-strong*	*Moderate-strong*	*Moderate-strong*	*Weak*	*Moderate-strong*
		**0.49**	**0.44**	0.32	**0.46**	**0.46**	**0.26**	**0.53**

*Note* Across each row for each EQ-5D-5L scale, the upper row represents the hypothesised pairwise correlation with the AQoL-6D scale while the bottom row displays the actual correlation coefficients obtained. Correlation coefficients < 0.4 and ≥ 0.4 indicate weak and moderate-strong correlations, respectively. All correlation coefficients are statistically significant with Bonferroni-corrected *p*-values either < 0.001 or *p* < 0.05^#^, except for those marked with * (*p* > 0.05). Bolded values indicate that the results were consistent with the pre-defined hypothesis

#### Known-group validity

Table 4 presents the results of hypothesis testing for between-group differences in AQoL-6D global score, as well as EQ-5D-5L metrics (as reference) and individual AQoL-6D dimension scores. Mann-Whitney U or Kruskal-Wallis H tests were utilised due to the non-normality of HRQoL data. It was observed that younger patients (aged < 40 years old) reported a significantly better average AQoL-6D global score than older patients (aged ≥ 40 years) (*p* = 0.049). These findings contrast with the literature-described relationship between age and HRQoL, where older patients typically report better HRQoL compared to younger age group [34]. Nonetheless, similar trend was observed with EQ-5D-5L-derived scores. In comparison to their male counterparts, female HF patients had lower global score, although statistical significance was not achieved (*p* = 0.257). As expected, patients in the NYHA class III-IV subgroup reported significantly lower AQoL-6D global scores compared to those in the NYHA class I-II subgroup (*p* < 0.001), with a notably large effect size (Cohen’s *d* = 0.8). Moreover, patients with a CCI reported significantly worse global scores compared to those with a lower CCI (*p* = 0.006). Compared to patients without comorbid diabetes, those with diabetes reported lower AQoL-6D global scores, although the difference was not statistically significant. Conversely, anaemic patients reported significantly worse AQoL-6D global scores compared to non-anaemic patients (*p* = 0.012). A significant and moderately large difference in the average AQoL-6D global score (*p* = 0.001, Cohen’s *d* = 0.75) was also observed between patients who needed high-dose diuretic therapy compared to those who did not. Altogether, five out of seven a priori hypotheses regarding between-group score differences were supported, except those related to age and sex. Nonetheless, the reference EQ-5D-5L metrics exhibited similar between-group differences as AQoL-6D global score, which further validated the concurrent and known-group validity of the Malay-AQoL-6D. Exploratory analyses of the associations between individual dimension scores and patient characteristics revealed that significant differences between patient groups were more frequently observed in the Independent Living and Relationship dimensions.

**Table 4:** Univariate analyses for known-group validation

Subgroup based on characteristic	*N* = 285	AQoL-6D*		EQ-5D-5L* (reference)		AQoL-6D dimension score* (exploratory)					
		Global score	HSUV	EQ-VAS	HSUV	Independent living	Relationships	Mental health	Coping	Pain	Senses
**Age**											
18–39	38	81 (12)	0.825 (0.163)	88 (12)	0.905 (0.156)	80 (17)	86 (21)	76 (18)	70 (15)	83 (17)	90 (8)
40–59	128	75 (15)	0.749 (0.210)	78 (18)	0.826 (0.195)	71 (22)	78 (22)	75 (20)	66 (23)	75 (19)	83 (11)
≥ 60	119	75 (14)	0.759 (0.188)	77 (17)	0.828 (0.182)	71 (22)	79 (18)	79 (18)	65 (21)	78 (19)	80 (12)
*p*-value^#^		**0.049**	0.152	**< 0.001**	**0.012**	0.070	**0.014**	0.362	0.526	0.070	**< 0.001**
Pairwise comparison with significant difference by Dunn’s test, *p*-value^#^		18–39 vs. 40–59, ***p* = 0.034** 18–39 vs. ≥60, ***p* = 0.030**	NS	18–39 vs. 40–59, ***p* = 0.003** 18–39 vs. ≥60, ***p* < 0.001**	18–39 vs. 40–59, ***p* = 0.009** 18–39 vs. ≥60, ***p* = 0.007**	NS	18–39 vs. 40–59, ***p* = 0.013** 18–39 vs. ≥60, ***p* = 0.007**	NS	NS	NS	18–39 vs. 40–59, ***p* < 0.001** 18–39 vs. ≥60, ***p* < 0.001**
For **18–39 vs. ≥40**: *p*-value^#^; Cohen’s *d* (95% CI)^†^		18–39 vs. ≥40, ***p* = 0.014; 0.40 (0.10, 0.71)**	18–39 vs. ≥40, *p* = 0.053; 0.37 (0.07, 0.66)	18–39 vs. ≥40, ***p* < 0.001; 0.62 (0.37–0.87)**	18–39 vs. ≥40, ***p* = 0.002; 0.42 (0.12, 0.72)**	18–39 vs. ≥40, *p* = 0.023; **0.42 (0.14**,** 0.70)**	18–39 vs. ≥40, ***p* = 0.004; 0.37 (0.02, 0.73)**	18–39 vs. ≥40, *p* = 0.703; −0.04 (−0.37, 0.28)	18–39 vs. ≥40, *p* = 0.335; 0.20 (−0.06, 0.46)	18–39 vs. ≥40, ***p* = 0.04; 0.33 (0.01, 0.66)**	18–39 vs. ≥40, ***p* < 0.001; 0.77 (0.50, 1.04)**
**Sex**											
Male	216	76 (14)	0.766 (0.199)	78 (17)	0.838 (0.193)	74 (21)	79 (20)	77 (20)	67 (22)	77 (19)	83 (11)
Female	69	74 (13)	0.754 (0.188)	82 (17)	0.833 (0.164)	68 (23)	80 (21)	75 (16)	64 (22)	78 (18)	83 (11)
*p*-value^#^		0.257	0.432	0.058	0.356	0.055	0.473	0.140	0.327	0.676	0.993
Cohen’s *d* (95% CI)^†^		0.14 (−0.12, 0.40)	0.06 (−0.20, 0.33)	−0.21 (−0.48, 0.054)	0.03 (−0.23, 0.29)	0.29 (−0.01, 0.58)	−0.07 (−0.35, 0.21)	0.13 (−0.12, 0.38)	0.13 (−0.13, 0.40)	−0.06 (−0.32, 0.20)	−0.03 (−0.28, 0.27)
**NYHA classification**											
I-II	257	77 (14)	0.778 (0.190)	80 (17)	0.851 (0.177)	74 (21)	80 (20)	77 (19)	67 (21)	78 (18)	83 (11)
III-IV	26	66 (15)	0.632 (0.211)	70 (16)	0.712 (0.223)	55 (21)	67 (22)	72 (22)	56 (23)	68 (22)	78 (14)
*p*-value^#^		**< 0.001**	**0.001**	**0.001**	**< 0.001**	**< 0.001**	**< 0.001**	0.2451	**0.008**	**0.013**	0.069
Cohen’s *d* (95% CI)^†^		**0.80** **(0.35**,** 1.24)**	**0.77** **(0.29–1.24)**	**0.59** **(0.17**,** 1.01)**	**0.77** **(0.26**,** 1.27)**	**0.94** **(0.50**,** 1.37)**	**0.66** **(0.21**,** 1.11)**	0.26 (−0.22, 0.74)	**0.53** **(0.08–0.98)**	**0.54** **(0.07**,** 1.01)**	0.55 (0.06, 1.03)
**Charlson comorbidity index (median = 4)**											
CCI ≤ 4	177	78 (14)	0.780 (0.193)	82 (16)	0.862 (0.168)	76 (20)	81 (19)	76 (19)	68 (21)	79 (18)	85 (11)
CCI > 4	108	73 (14)	0.736 (0.199)	74 (18)	0.796 (0.207)	66 (23)	76 (22)	78 (18)	63 (23)	75 (19)	80 (11)
*p*-value^#^		**0.006**	0.054	**< 0.001**	**0.005**	**0.001**	**0.021**	0.444	0.063	0.096	**< 0.001**
Cohen’s *d* (95% CI)^†^		**0.33** **(0.08**,** 0.57)**	0.23 (−0.02, 0.47)	**0.51** **(0.25**,** 0.77)**	0.36 (0.11–0.62)	**0.46** **(0.21**,** 0.72)**	**0.28** **(0.03**,** 0.54)**	−0.01 (−0.34, 0.15)	0.23 (−0.02, 0.48)	0.21 (−0.05, 0.46)	**0.42** **(0.17**,** 0.68)**
**Diabetes mellitus**											
Yes	119	74 (15)	0.775 (0.192)	80 (17)	0.826 (0.176)	67 (24)	77 (23)	77 (18)	64 (23)	76 (20)	82 (11)
No	166	77 (14)	0.746 (0.201)	77 (17)	0.845 (0.193)	76 (19)	80 (19)	76 (20)	67 (21)	78 (18)	84 (11)
*p*-value^#^		0.067	0.249	0.053	0.134	**0.004**	0.624	0.920	0.250	0.380	0.092
Cohen’s *d* (95% CI)^†^		0.23 (−0.02, 0.48)	0.15 (−0.09, 0.39)	0.18 (−0.05, 0.41)	0.11 (−0.14, 0.36)	**0.41** **(0.17**,** 0.65)**	0.15 (−0.09, 0.39)	−0.04 (−0.27, 0.19)	0.15 (−0.11, 0.40)	0.12 (−0.12, 0.37)	0.16 (−0.08, 0.40)
**Anaemia** ^**§**^											
Yes	34	70 (16)	0.773 (0.192)	73 (17)	0.760 (0.212)	61 (26)	70 (24)	74 (19)	59 (24)	74 (20)	81 (14)
No	251	77 (14)	0.693 (0.218)	80 (17)	0.848 (0.180)	74 (21)	80 (20)	77 (19)	67 (21)	78 (19)	83 (11)
*p*-value^#^		**0.012**	**0.037**	**0.009**	**0.007**	**0.003**	**0.003**	0.402	0.092	0.143	0.392
Cohen’s *d* (95% CI)^†^		**0.51 (0.11**,** 0.92)**	**0.41** **(0.18**,** 0.80)**	**0.40** **(0.03**,** 0.74)**	**0.48** **(0.06**,** 0.90)**	**0.62** **(0.19**,** 1.06)**	**0.53** **(0.11**,** 0.94)**	0.15 (−0.22, 0.51)	0.34 (−0.08, 0.76)	0.30 (−0.09, 0.70)	0.24 (−0.20, 0.69)
**High-dose diuretic therapy** ^**‡**^											
Yes	17	66 (15)	0.615 (0.210)	70 (18)	0.753 (0.188)	59 (26)	71 (24)	64 (20)	53 (23)	66 (25)	82 (10)
No	268	76 (14)	0.773 (0.192)	80 (17)	0.842 (0.185)	73 (21)	80 (20)	77 (19)	67 (21)	78 (18)	83 (11)
*p*-value^#^		**0.001**	**0.003**	**0.012**	**0.023**	**0.026**	0.075	**0.009**	**0.012**	0.051	0.549
Cohen’s *d* (95% CI)^†^		**0.75 (0.20–1.30)**	**0.82** **(0.26**,** 1.38)**	**0.58** **(0.04**,** 1.11)**	**0.48** **(−0.05**,** 1.02)**	**0.65** **(0.25**,** 1.28)**	0.44 (−0.15, 1.04)	**0.69** **(0.14**,** 1.23)**	**0.61** **(0.05**,** 1.17)**	0.65 (−6E-5, 1.30)	0.05 (−0.38, 0.48)

*AQoL-6D* the Assessment of Quality of Life—6 Dimensions instrument, *Cl* confidence interval, *EQ-5D-5L* the five-level version of the EuroQoL 5 dimensions instrument, *EQ-VAS* EuroQol visual analogue scale, *HSUV* health state utility value, *NYHA* New York Heart Association functional class, *NS* non-significant

*Mean (SD)

^#^Bonferroni-corrected *p*values were reported: bold values represent statistically significant differences (*p* < 0.05) based on either Mann-Whitney U (2 groups) or Kruskal-Wallis H test (≥ 3 groups)

^†^Bootstrapped 95% confidence intervals (1000 repetitions) were reported for effect sizes. Effect sizes and corresponding Cohen’s *d* values: small effect (0.2-<0.4), moderate effect (0.4-<0.8), and large effect (≥ 0.8)

^§^Defined as haemoglobin level < 12 g/dL in women or < 13 g/dL in men

^‡^Defined as frusemide equivalent dose ≥ 80 mg per day or combination of two non-MRA diuretics (e.g., a loop diuretic + a thiazide-like diuretic)

#### Robustness of validation results

Little’s MCAR test (χ^2^ distance = 44.9, *df* = 43, and *p* = 0.392) confirmed that the missing values met the MCAR assumption. The validation results remained robust across different missing data techniques and sample subsets that excluded proxy-reports or responses requiring assistance (refer to Online Supplementary Section 7, S7.3 and S7.4 for more details).

## Discussion

This study translated and cross-adapted the AQoL-6D into Malay and evaluated its psychometric properties (acceptability, reliability, and validity) within the context of the local HF population. While various translations of the instrument exist [12], prior research using the AQoL-6D was predominantly confined to the Australian context. This study not only developed a Malay version of the instrument but also validated its psychometric properties. These findings hold the promise for improving the AQoL-6D’s utility not only for HF patients in Malaysia but also for other populations in other Malay-speaking regions.

The cross-cultural validity of the Malay-AQoL-6D was diligently ensured through a rigorous cross-cultural adaptation process. The involvement of an expert panel and target users representing different sociodemographic backgrounds was crucial to ensure that the process adequately accounted for Malaysia’s diverse, multireligious society. The choice of words in the Malay-AQoL-6D held particular significance to ensure readability, understandability, and cultural sensitivity. A notable example is that the place of worship in item-7 of the AQoL-6D was thoughtfully revised to ‘*tempat beribadat*’ instead of originally proposed “*gereja*’, which translates to church in Malay.

The Malay-AQoL-6D demonstrated good psychometric performance, consistent with the findings from prior studies [7, 9]. The instrument was well-received, showing low levels of missingness and minimal ceiling effects. The validity assessments indicated strong correlations between Malay-AQoL-6D items and their respective dimension scores, which, in turn, showed a strong correlation with the overall construct of HRQoL as measured by the AQoL-6D. The concurrent validity of the Malay-AQoL-6D was supported by the strong agreement observed with the EQ-5D-5L. Furthermore, evidence supporting known-group validity of the Malay-AQoL-6D provides assurance that the instrument indeed possesses good sensitivity in distinguishing between patient groups known to differ in their sociodemographic and clinical characteristics.

The inter-relatedness among items within the same health dimensions, assessed through Cronbach’s alpha and MacDonald’s omega values, were generally satisfactory, except for the items within the Senses dimension, which exhibited poor item reliabilities (Cronbach’s alpha: 0.36) and loadings (ranging from 0.34 to 0.52). These findings were consistent with previous studies [7, 9], and reaffirm the notion that sensory impairments related to seeing, hearing, and communication may not necessarily coexist. Nevertheless, these items collectively contributed to the HRQoL construct, as evidenced by a loading-coefficient of 0.70 between the Senses dimension and the AQoL-6D. While it might be tempting to treat them as distinct dimensions, such an approach did not improve loadings and instead resulted in a less favourable model fit.

Allen et al. (2013) [9] previously proposed the existence of two higher-order factors or super-dimensions—physical and psychological—within the AQoL-6D structure. This suggested framework linked the Coping and Mental Health dimensions to the psychological factor, while grouping the remaining four dimensions into the physical factor. However, Maxwell et al. (2016) [10], also drawing on data from general populations, contested this notion. Their analyses demonstrated that the AQoL-6D did not have sufficient dimensions to be further delineated into physical and psycho-social super-dimensions, which could only be possible with the inclusion of two additional dimensions of Happiness and Self-Worth, as shown in the AQoL—8 dimensions (AQoL-8D). Besides, Maxwell et al. placed the Relationships dimension under Psycho-social, rather than the Physical super-dimension. The findings of this study closely aligned with Maxwell et al., indicating that the six dimensions are not necessarily demarcated by two higher-order factors (see Online Supplementary Section 6, S6.2) for further details).

As observed in a previous study of individuals at risk of osteoporotic fracture [43], AQoL-6D-derived HSUVs were lower than EQ-5D-5L HSUVs. The AQoL-6D, designed to assess *handicap*, exhibits higher sensitivity in detecting limitations across physical, mental, and social functioning, even among individuals who may appear physically well [7]. In the current cohort, where > 90% reported either no or slight physical limitations (NYHA class I-II), AQoL-6D provided a more comprehensive HRQoL evaluation compared to EQ-5D-5L. It revealed unaddressed health issues, particularly through its Relationships and Coping dimensions. While EQ-5D-5L might have indicated the presence of issues in these dimensions via its Mobility and Usual Activities dimensions, as suggested by strong pairwise correlations among the dimensions, AQoL-6D precisely identified the exact problems. Nevertheless, the mean difference in HSUVs between the two instruments was small and close to the minimally important difference estimated for the Malaysian EQ-5D-5L value set by Henry et al., at 0.072 (0.053–0.088) [44]. Applying this threshold, about 43% of cases within this cohort reported comparable HSUVs using both instruments. While complete agreement between the two instruments is not expected due to inherent differences in dimension spectrums and the use of value sets derived from two culturally distinct populations, the magnitude of differences in HSUVs between patient groups was highly comparable between the two instruments. These findings not only supported the concurrent and known-group validity of the Malay-AQoL-6D but also suggested their interchangeability, although more studies in different patient groups are needed to test this hypothesis.

Although mean imputation for missing ordinal values is generally discouraged due to its potential to underestimate standard errors, it has been integrated into the AQoL-6D utility scoring algorithm [37]. The algorithm automatically replaces missing ordinal responses with the rounded average responses from the other items within the same dimensions, provided that only one item within each dimension is missing. The current study demonstrated that mean imputation, which is less time-consuming and laborious, may yield comparable results to less biased and more acceptable techniques including RFIML and multiple imputation [36] when the proportions of missing data are low.

The current study has several limitations. First, while HCPs were involved in the forward translation process to ensure clinical equivalency of the source and target versions, there was a deviation from the guideline recommendation to involve a language professional. However, the final Malay-AQoL-6D version was deemed to adequately reflect the language used by the Malaysian people after rounds of expert panel discussion, pre-testing and content validation. Second, the responsiveness of the Malay-AQoL-6D was not explored due to the cross-sectional nature of this study. Third, only a small percentage (5%) of respondents participated in the test-retest reliability assessment because the repeat survey was not initially planned for the HRQoL-HF-MOH survey but was only undertaken near the end of recruitment. Moreover, mixed data collection modes (paper and electronic format) of the Malay-AQoL-6D in the initial and repeat survey were used to reduce respondent burden. This approach might have discouraged some test-retest respondents from participating and introduced potential measurement error. Therefore, caution is needed when interpreting the test-retest reliability results, as the equivalence of the paper- and electronic forms of the instrument is not yet established. Fourth, a well-established HF-specific HRQoL measure such as the Kansas City Cardiomyopathy Questionnaire [45] was not used in the current study for validity assessments of the Malay-AQoL-6D. Responsiveness, test-retest reliability, and validity could be confirmed in a future longitudinal study where the Malay-AQoL-6D, alongside a HF-specific measure, is administered at set timepoints, either by the same mode or through a randomised crossover equivalence design [46]. Lastly, the study observed a high rate of assistance required to fill out the survey (20.7%). This finding may explain the relatively low number of item non-responses but also suggested that a significant number of respondents might find either filling out the survey itself (including sections on sociodemographic profile, EQ-5D-5L and AQoL-6D) or certain items in the Malay-AQoL-6D challenging. The exact causes were not known since the survey did not systematically record respondents’ opinions, nor track the time needed to complete the questionnaire. Feedback from data collectors indicated variations in the time taken, with some respondents taking longer to complete the survey than others. Common reasons for requiring assistance included limited sight to read the questionnaires printed on A4-sized papers and reluctance to read through the questions independently. In such cases, the data collectors had to read the questions aloud, and thus, assistance was deemed necessary. In a future study, respondents can be asked for their opinion on the Malay-AQoL-6D separately, and the time needed to answer can be tracked. Additionally, better respondent accessibility can be achieved by offering large print and audio-computer-assisted self-interview options to those who need them. The respondent’s feedback can also be helpful in confirming the reasons for the relatively high missing responses for item-5 (*intimacy*), which might be caused by either sexual inactivity or respondent preference ‘not to say’ due to the conservative sexual attitudes of local population [47]. Despite these limitations, this study has successfully developed the Malay-AQoL-6D, laying the foundation for future efforts in AQoL-6D valuation to create the Malaysian value set that reflects the preferences of local people. Such endeavours can facilitate the broader adoption of this valuable HRQoL measure.

## Conclusion

This study developed the Malay-AQoL-6D and demonstrated that it may be acceptable, reliable, and valid for evaluating HRQoL among the local HF population. The Malay-AQoL-6D appears to be a suitable alternative to EQ-5D-5L, providing more granular information to identify unmet needs and showing better sensitivity in discerning health states among less physically unwell patients. The findings also indicated the feasibility of routine HRQoL assessment in HF clinics, paving the way ahead for its local implementation, which has the potential to improve outcomes of HF patients, as consistently observed in previous research. Future research is needed to confirm the test-retest reliability and responsiveness of the Malay-AQoL-6D, and validity of the Malay-AQoL-6D against other generic or disease-specific instruments. The availability of the Malay-AQoL-6D facilitates these research endeavours and promotes the wider utilisation of this HRQoL measure among HF patients and beyond.

### Electronic supplementary material

Below is the link to the electronic supplementary material.

Supplementary Material 1

## Data Availability

The dataset used and/or analysed during the current study are available from the corresponding author on reasonable request.

## Abbreviations

AQoL-6D: Assessment of Quality of Life—6 Dimensions instrument
Ave-CVI: Average content validity index
CCI: Charlson comorbidity index
CFA: Confirmatory factor analysis
CFI: Comparative fix index
CI: Confidence interval
CKD: Chronic kidney disease
COSMIN: Consensus-based Standards for the Selection of Health Measurement Instruments
CVI: Content validity index
df: Degree of freedom
DM: Diabetes mellitus
EQ-5D-5L: Five-level version of the EuroQoL 5 dimensions instrument
EQ-VAS: EuroQol visual analogue scale
HCPs: Healthcare professional
HF: Heart failure
HRQoL-HF-MOH: Health-related Quality of Life among Chronic Heart failure patients treated in Malaysian Ministry of Health public centres study
HRQoL: Health-related quality-of-life
HSUV: Health state utility values
I-CVI: Item-level content validity index
ICC: Intraclass correlation coefficient
ISPOR: The Professional Society for Health Economics and Outcomes Research
LOA: Limits of agreement
LVEF: Left ventricular ejection fraction
Malay-AQoL-6D: The Malay version of the Assessment of Quality of Life—6 Dimensions
MCAR: Missing completely at random
MI-MVN: Multiple multivariate normal imputation
MI: Multiple imputation
ML: Maximum likelihood estimation
NYHA: New York Heart Association functional class
pclose: Close fit *p*-value
RFIML: Robust full information maximum likelihood
RMSEA: Root mean square error of approximation
SB: Satorra-Bentler correction
SD: Standard deviation
SRMR: Standardised root-mean-square residual
TLI: Tucker-Lewis index
