# Supplementary material for: Translation, cross-cultural adaptation, and psychometric validation of the Malay version of the Assessment of Quality of Life—6 Dimensions (Malay-AQoL-6D) instrument among Malaysians living with chronic heart failure
Source: J Patient Rep Outcomes. 2024 Jul 25;8:79. doi: 10.1186/s41687-024-00763-3 (PMC11272755; doi:10.1186/s41687-024-00763-3)
Supplement: Supplementary file 1 — Supplementary Material 1 [file 41687_2024_763_MOESM1_ESM.docx]

# Additional File 1: Supplementary Information

**Title**

Translation, cross-cultural adaptation, and psychometric validation of the Malay version of the Assessment of Quality of Life – 6 Dimensions (Malay-AQoL-6D) instrument

**Journal**

Journal of Patient-Reported Outcomes

**Section 1**

**S1.1** Comparison between the AQoL-6D and the EQ-5D-5L instruments

|  | **AQoL-6D** | **EQ-5D-5L** |
| --- | --- | --- |
| Dimension and (number of items) | 6 dimensions   - Independent living (4 items) - Relationships (3 items) - Mental health (4 items) - Coping (3 items) - Pain (3 items) - Senses (3 items) | 5 dimensions   - Mobility (1 item) - Self-care (1 item) - Usual activities (1 item) - Pain/discomfort (1 item) - Anxiety/depression (1 item) |
| Response level | 4 to 6 levels | 5 levels |
| Alignment between two instruments in health dimensions (1) | **Dimension 1: Independent living**  Q2: getting around  Q3: mobility | Q1: Mobility |
|  | Q4: self-care | Q2: Self-care |
|  | Q1: household tasks (activities of daily living) | Q3: Usual activities |
|  | **Dimension 5: Pain**  Q15: frequency of pain  Q16: degree of pain  Q17: interference with usual activities caused by pain | Q4: Pain/discomfort |
|  | **Dimension 3: Mental health**  Q8: despair*  Q9: worry*  Q10: sadness*  Q11: agitation/tranquillity* | Q5: Anxiety/depression* |
|  | **Dimension 2: Relationships**  Q5: friendships/intimacy  Q6: family role  Q7: community/social function | Not assessed |
|  | **Dimension 4: Coping**  Q12: energy level/vitality  Q13: being in control  Q14: coping with problems | Not assessed |
|  | **Dimension 6: Senses**  Q18: seeing  Q19: hearing  Q20: communication | Not assessed |
| Valuation set | Based on time-trade-off (TTO) and person trade-off (PTO) interviews done with 361 randomly selected members of the public in Australia | Derived using standardised protocol and the EuroQoL Valuation Technology (EQ-VT), which included composite TTO and discrete choice experiment (DCE) task. |
| Minimum and maximum utility value possible | -0.044 to 1.000 | -0.442 to 1.000 |
| Minimally important difference (MID) | 0.06 or effect size ≥0.13 (2) | - 0.072 based on Malaysian value set for EQ-5D-5L using simulation-based, instrument-defined approach (3) - 0.074 (4) based on UK value set for EQ-5D-3L |

The five dimensions of the EQ-5D-5L were also included in the AQoL-6D instrument, with some notable differences, with the latter providing more HRQoL information. A key difference emerges in the AQoL-6D approach to the Mental Health dimension. While both instruments address anxiety and depression, the AQoL-6D focuses on the frequency (“how often”) of mental problems rather than their severity (“how severe”) in the EQ-5D-5L instrument. Moreover, the Pain dimension differs between two instruments. The EQ-5D-5L assesses the degree of pain or discomfort, whereas the AQoL-6D also delves further into the frequency of severe pain and how pain interferes with daily activities. Unique to the AQoL-6D instrument are three additional health dimensions: Relationships dimension encompassing intimacy, family relationship, and social functioning; Coping, which examines vitality, coping abilities, and the sense of control over one’s life; and Senses, which evaluates hearing, vision, and communication abilities with others.

**S1.2.1** Original English AQoL-6D

|  |  |
| --- | --- |
|    |  |

**S1.2.2** Final Malay-AQoL-6D

|  |  |
| --- | --- |
|  |  |
|  |  |

**Section 2**: Issues encountered during the cross-cultural adaptation process and resolution

| **Item number** | **Original English version** | **Problem** | **Resolution** |
| --- | --- | --- | --- |
| Synthesis | | | |
| 18 | Response level 5:  *I only see general shapes. I need a* ***guide*** *to move around.* | Understandability: The word ‘guide’ was originally translated into ‘*pemandu’* and ‘*bantuan’* by the two forward-translators. | The word ‘*bantuan’* was chosen as it was deemed easier to understand by the general public. |
| 20 | *How well do you communicate with others (talking,* ***signing****, texting, being understood by others, and understanding them)* | Misinterpretation: The word ‘signing’ was misinterpreted as writing one’s name on a document as an agreement and erroneously translated as ‘*menandatangani’.* | The intended interpretation is ‘using sign language to communicate’ in this case, which translates to “*menggunakan bahasa isyarat*” |
| Harmonisation exercise and pre-testing | | | |
| 2 | Response level 1:  ***Getting around*** *is enjoyable and easy.* | Readability: The phrase ‘*getting around’* was originally translated as *‘ke sana ke mari’*, which may not be readable. | The translation was revised to ‘*ke mana-mana’* to improve readability |
|  | Response level 2:  *I have no difficulty* ***getting around*** *outside my place of residence.* |  |  |
| 4 | Response level 5:  *I cannot do these tasks by myself* ***at all.*** | Readability: The phrase ‘at all’ was translated as ‘…*sama sekali’*, which may not give sufficient emphasis. | The translation was revised to ‘*saya langsung tidak dapat…’* to improve readability. |
| 13 | *How often do you feel in control of your life?* | Readability: The phrase ‘*in control of your life’* was originally translated as ‘*kehidupan anda berada dalam kawalan’* | The translation was revised to ‘*kehidupan anda berada dalam kawalan kendiri’* to improve readability |
| 7 | *Does your health affect your role in your community (e.g., residential, sporting,* ***church****, or cultural groups)?* | Cultural sensitivity: The translated version for the word ‘church’ may not be relevant to the country’s diverse, multireligious society. | The word ‘*tempat beribadat’* was used instead, which means ‘place of worship’, which include mosques for Muslims, temples for Buddhists and Hindus, and churches for Christians. |
| 18 | *Response level 4:*  *I have a lot of difficulty seeing things. My vision is blurred.* ***I can see just enough to get by with*** | Understandability: The English phrase “just enough to get by with” does not have an equivalent in Malay, and its direct translation, “*sekadar cukup untuk bertahan”* may not be understood and could be interpreted in different ways. | The Chinese version uses ‘我只能看到触手可及的物体’, which means ‘I can only see objects within reach’. This was considered easier to understand and less ambiguous, and was therefore used to inform the Malay version, i.e., “*Saya hanya dapat melihat objek dalam jarak yang dapat disentuh”.* |

**Section 3**: Content Validation

**S3.1** Adapted version of Simon and White’s Validation Rubric for Expert Panel (VREP) used in content validation of the pre-final Malay-AQoL-6D (5). Twelve panellists were required to independently assess the Malay-AQoL-6D instrument against eleven pre-defined criteria using a 4-point rating scale presented in a validation rubric form.

| **Criteria** | **Operational Definitions** | **Score**  **1=Not Acceptable** (major modifications needed)  **2=Below Expectations** (some modifications needed)  **3=Meets Expectations** (no modifications needed but could be improved with minor changes)  **4=Exceeds Expectations** (no modifications needed) | | | | **Questions NOT meeting standard**  **(List page and question number) and need to be revised.**  ***Please use the comments and suggestions section to recommend revisions.*** |
| --- | --- | --- | --- | --- | --- | --- |
|  |  | 1 | 2 | 3 | 4 |  |
| 1. **Clarity** | - The questions are direct and specific. - Only one question is asked at a time. - The participants can understand what is being asked. - There are no *double-barrelled* questions (two questions in one). |  |  |  |  |  |
| 1. **Wordiness** | - Questions are concise. - There are no unnecessary words. |  |  |  |  |  |
| 1. **Negative Wording** | - Questions are asked using the affirmative (e.g., Instead of asking, “Which methods are not used?”, the researcher asks, “Which methods *are* used?”) |  |  |  |  |  |
| 1. **Overlapping Responses** | - No response covers more than one choice. - All possibilities are considered. - There are no ambiguous questions. |  |  |  |  |  |
| 1. **Balance** | - The questions are unbiased and do not lead the participants to a response. The questions are asked using a neutral tone. |  |  |  |  |  |
| 1. **Use of Jargon** | - The terms used are understandable by the target population. - There are no clichés or hyperbole in the wording of the questions. |  |  |  |  |  |
| 1. **Appropriateness of Responses Listed** | - The choices listed allow participants to respond appropriately. - The responses apply to all situations or offer a way for those to respond with unique situations. |  |  |  |  |  |
| 1. **Use of Technical Language** | - The use of technical language is minimal and appropriate. - All acronyms are defined. |  |  |  |  |  |
| 1. **Application to Praxis** | - The questions asked relate to the daily practices or expertise of the potential participants. |  |  |  |  |  |
| 1. **Relationship to Problem** | - The questions are sufficient to resolve the problem in the study - The questions are sufficient to answer the research questions. - The questions are sufficient to obtain the purpose of the study. |  |  |  |  |  |
| 1. **Measure of Construct: Health-related quality of life** | - The survey adequately measures this construct, which includes 6 dimensions (Independent Living, Mental Health, Coping, Relationships, Pain, and Senses) |  |  |  |  |  |

| **Comments and suggestions** |
| --- |
|  |

**S3.2** Calculation of content validity indices (CVI)

|  | **Expert and rating scale used** (1= not acceptable; 2 = below expectations; 3 = meets expectations; 4 = exceeds expectations) | | | | | | | | | | | | **No. of agreements** | **Item-CVI** |
| --- | --- | --- | --- | --- | --- | --- | --- | --- | --- | --- | --- | --- | --- | --- |
| **Criterion**  **or item**  **Expert** | **Expert 1** | **Expert 2** | **Expert 3** | **Expert 4** | **Expert 5** | **Expert 6** | **Expert 7** | **Expert 8** | **Expert 9** | **Expert 10** | **Expert 11** | **Expert 12** |  |  |
| 1. Clarity | 4 | 3 | 4 | 4 | 3 | 4 | 3 | 3 | 4 | 4 | 4 | 4 | 12 | 1.00 |
| 1. Wordiness | 3 | 3 | 3 | 4 | 3 | 3 | 3 | 2 | 4 | 4 | 3 | 2 | 10 | 0.83 |
| 1. Negative wording | 4 | 4 | 4 | 4 | 3 | 4 | 2 | 4 | 4 | 4 | 4 | 4 | 11 | 0.92 |
| 1. Overlapping responses | 4 | 3 | 4 | 3 | 3 | 4 | 3 | 4 | 4 | 4 | 4 | 3 | 12 | 1.00 |
| 1. Balance | 4 | 4 | 4 | 4 | 3 | 4 | 3 | 4 | 4 | 4 | 4 | 3 | 12 | 1.00 |
| 1. Use of jargon | 4 | 3 | 4 | 3 | 3 | 4 | 3 | 4 | 4 | 4 | 4 | 4 | 12 | 1.00 |
| 1. Appropriateness of responses | 3 | 3 | 4 | 4 | 3 | 3 | 3 | 4 | 4 | 4 | 3 | 3 | 12 | 1.00 |
| 1. Use of technical language | 4 | 3 | 4 | 3 | 3 | 3 | 3 | 3 | 4 | 4 | 3 | 4 | 12 | 1.00 |
| 1. Application to praxis | 4 | 3 | 4 | 4 | 3 | 4 | 3 | 4 | 4 | 4 | 4 | 4 | 12 | 1.00 |
| 1. Relationship to problem | 4 | 3 | 4 | 4 | 3 | 4 | 3 | 3 | 4 | 4 | 4 | 4 | 12 | 1.00 |
| 1. Measure of HRQoL construct | 4 | 4 | 4 | 4 | 3 | 3 | 3 | 4 | 4 | 4 | 4 | 4 | 12 | 1.00 |
| Average-CVI = (Σ Item-CVI/total no. of items) | | | | | | | | | | | | | | 0.98 |

**Section 4:** Recruitment details of the HRQoL-HF-MOH study

**S4.1** Flow chart of patient recruitment and inclusion in the analysis.


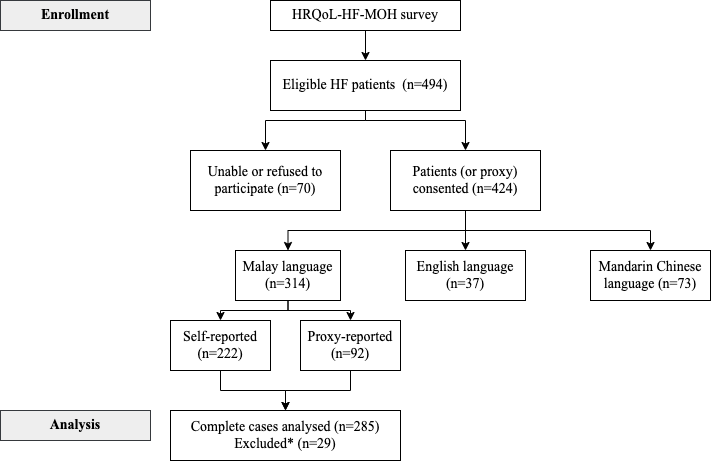


*Patients excluded from the base-case analysis due to one or more item noncompletions on the Malay-AQoL-6D (missing rate: 9.2%). Several missing data techniques were carried out to impute the missing values, and the validation results using the full dataset (n=314) were examined in sensitivity analyses.

HRQoL-HF-MOH = health-related quality of life among chronic heart failure patients treated in Ministry of Health (Malaysia) specialist centres study; HF = heart failure

**S4.2** Participation rates across study sites

| **Region** | **State** | **Centre** | **Number of subjects screened** | **Refusal or incapability to participate** | **Number of subjects recruited** | **Participation rate (%)** |
| --- | --- | --- | --- | --- | --- | --- |
| East coast | Pahang | Hospital Tengku Ampuan Afzan (HTAA) | 36 | 7 | 29 | 80.6 |
| Sabah | Sabah | Hospital Duchess of Kent (HDOK) | 11 | 0 | 11 | 100.0 |
| Sabah | Sabah | Hospital Queen Elizabeth II (HQE2) | 127 | 13 | 114 | 89.8 |
| Sarawak | Sarawak | Hospital Umum Sarawak (HUS) | 92 | 9 | 83 | 90.2 |
| West coast | Penang | Hospital Pulau Pinang (HPP) | 128 | 25 | 103 | 80.5 |
| West coast | Perak | Hospital Seri Manjung (HSM) | 69 | 5 | 64 | 92.7 |
| West coast | Perak | Hospital Teluk Intan (HTI) | 31 | 11 | 20 | 64.5 |
|  | Total | | 494 | 70 | 424 | 85.8 |

**S4.3** Distribution of different HRQoL scores

AQoL-6D: the Assessment of Quality of Life – 6 Dimensions instrument; EQ-5D-5L = the five-level version of the EuroQoL 5 dimensions instrument; EQ-VAS = EuroQol visual analogue scale

**Section 5:** Reliability of the Malay-AQoL-6D

**S5.1** Internal consistency (n=285)

| **AQoL-6D dimension** | **Item** | **Cronbach’s alpha (≥0.7)** | **Item-rest correlation (≥0.2)** | **MacDonald’s omega (≥0.7)** |
| --- | --- | --- | --- | --- |
| Independent Living | 1 | 0.89 | 0.77 | 0.90 |
|  | 2 |  | 0.80 |  |
|  | 3 |  | 0.80 |  |
|  | 4 |  | 0.75 |  |
| Relationships | 5 | 0.74 | 0.50 | 0.77 |
|  | 6 |  | 0.67 |  |
|  | 7 |  | 0.57 |  |
| Mental Health | 8 | 0.87 | 0.71 | 0.87 |
|  | 9 |  | 0.81 |  |
|  | 10 |  | 0.75 |  |
|  | 11 |  | 0.63 |  |
| Coping | 12 | 0.72 | 0.53 | 0.72 |
|  | 13 |  | 0.51 |  |
|  | 14 |  | 0.56 |  |
| Pain | 15 | 0.80 | 0.62 | 0.82 |
|  | 16 |  | 0.68 |  |
|  | 17 |  | 0.70 |  |
| Senses | 18 | 0.36 | 0.19 | 0.44 |
|  | 19 |  | 0.28 |  |
|  | 20 |  | 0.22 |  |

**S5.2** Test-retest reliability (n=15)

Fifteen respondents completed the Malay-AQoL-6D twice at an interval of 7-14 days. The intraclass correlation coefficients (and 95% confidence intervals) associated with each AQoL-6D dimension score were shown below.

| **Intraclass correlation coefficient*** | **Independent Living** | **Relation-ships** | **Mental Health** | **Coping** | **Pain** | **Senses** | **Global score** |
| --- | --- | --- | --- | --- | --- | --- | --- |
| Individual | 0.647  (0.213, 0.867) | 0.809  (0.526, 0.931) | 0.806  (0.500, 0.931) | 0.837  (0.579, 0.942) | 0.781  (0.468, 0.921) | 0.905  (0.742, 0.967) | 0.875  (0.659, 0.957) |
| Average | 0.786  (0.351, 0.929) | 0.894  (0.689, 0.964) | 0.893  (0.667, 0.964) | 0.911  (0.734, 0.970) | 0.877  (0.637, 0.959) | 0.950  (0.852, 0.983) | 0.934  (0.794, 0.978) |
| p-value for significance | 0.001 | <0.001 | <0.001 | <0.001 | <0.001 | <0.001 | <0.001 |

*Two-way mixed effects model, based on an absolute agreement definition

**Section 6**: Construct validity

**S6.1** Two-level, six-factor model structure of the Malay-AQoL-6D, estimated using maximum likelihood method with Satorra-Bentler correction (ML-SB) (n=285).

| **HRQoL** | **Between AQoL-6D and dimension** | | | | | | **AQoL-6D dimension** | **Item** | **Between dimension and items** | | | | | |
| --- | --- | --- | --- | --- | --- | --- | --- | --- | --- | --- | --- | --- | --- | --- |
|  | **Unstandardised** | | | **Standardised** | | |  |  | **Unstandardised** | | | **Standardised** | | |
|  | **B** | **SE** | **ϵ** | **β** | **SE** | **ϵ** |  |  | **B** | **SE** | **ϵ** | **β** | **SE** | **ϵ** |
| **AQoL-6D** | 1.00 | - | 0.25 | 0.82 | 0.03 | 0.33 | **Independent Living** | **1** | 1.00 | - | 0.34 | 0.83 | 0.02 | 0.31 |
|  |  |  |  |  |  |  |  | **2** | 1.31 | 0.07 | 0.53 | 0.84 | 0.02 | 0.29 |
|  |  |  |  |  |  |  |  | **3** | 1.11 | 0.06 | 0.32 | 0.86 | 0.02 | 0.26 |
|  |  |  |  |  |  |  |  | **4** | 0.78 | 0.04 | 0.25 | 0.81 | 0.02 | 0.35 |
|  | 0.69 | 0.10 | 0.07 | 0.89 | 0.03 | 0.21 | **Relationships** | **5** | 1.00 | - | 0.51 | 0.62 | 0.05 | 0.62 |
|  |  |  |  |  |  |  |  | **6** | 0.88 | 0.10 | 0.18 | 0.76 | 0.03 | 0.43 |
|  |  |  |  |  |  |  |  | **7** | 1.35 | 0.17 | 0.35 | 0.78 | 0.04 | 0.38 |
|  | 0.64 | 0.07 | 0.29 | 0.65 | 0.04 | 0.58 | **Mental health** | **8** | 1 | - | 0.31 | 0.78 | 0.04 | 0.39 |
|  |  |  |  |  |  |  |  | **9** | 1.13 | 0.07 | 0.17 | 0.89 | 0.03 | 0.21 |
|  |  |  |  |  |  |  |  | **10** | 1.09 | 0.08 | 0.30 | 0.81 | 0.03 | 0.34 |
|  |  |  |  |  |  |  |  | **11** | 0.79 | 0.07 | 0.34 | 0.69 | 0.03 | 0.52 |
|  | 1.03 | 0.08 | 0.11 | 0.91 | 0.04 | 0.17 | **Coping** | **12** | 1 | - | 0.38 | 0.80 | 0.03 | 0.36 |
|  |  |  |  |  |  |  |  | **13** | 0.65 | 0.07 | 0.69 | 0.54 | 0.06 | 0.71 |
|  |  |  |  |  |  |  |  | **14** | 0.76 | 0.07 | 0.54 | 0.65 | 0.04 | 0.58 |
|  | 0.52 | 0.06 | 0.04 | 0.74 | 0.05 | 0.45 | **Pain** | **15** | 1 | - | 0.27 | 0.70 | 0.04 | 0.51 |
|  |  |  |  |  |  |  |  | **16** | 0.84 | 0.09 | 0.14 | 0.75 | 0.03 | 0.44 |
|  |  |  |  |  |  |  |  | **17** | 1.53 | 0.14 | 0.20 | 0.87 | 0.03 | 0.25 |
|  | 0.28 | 0.08 | 0.52 | 0.70 | 0.08 | 0.51 | **Senses** | **18** | 1 | - | 0.66 | 0.34 | 0.08 | 0.89 |
|  |  |  |  |  |  |  |  | **19** | 1.45 | 0.44 | 0.49 | 0.52 | 0.08 | 0.73 |
|  |  |  |  |  |  |  |  | **20** | 0.62 | 0.20 | 0.12 | 0.45 | 0.08 | 0.79 |

Note: All coefficients have p-values <0.001.

B = unstandardised coefficient; β = standardized coefficient; ϵ = error term (residual); SE = standard error

**S6.2** Confirmatory factor analysis of an alternative two super-dimension structure (physical and psychological) in the AQoL-6D, as proposed by Allen et al. (6)

Note: The AQoL-6D generates six dimension scores as follows: Independent Living (denoted as dim_ind_living), Relationship (denoted as dim_relation), Mental Health (denoted as dim_mental), Coping (denoted as dim_coping), Pain (denoted as dim_pain), and Senses (denoted as dim_senses). Standardised factor-loadings were reported, all having p-value <0.001. ϵ denotes the error terms. From the left, the four sets of numbers respectively represent: the error terms on each dimension, the loadings between the dimension scores and the proposed super-dimensions, and the variance of the super-dimensions, both constrained to 1, and lastly, the covariance between the super-dimensions, which approaches 1, indicating no distinction between them.

**Section 7**: Missing data analysis, missing data techniques, and robustness analyses

**S7.1** Missing data analysis (total n=314)

| **AQoL-6D dimension** | **Item** | **Non-response – no. (%)** | | **Possible reason for non-response** |
| --- | --- | --- | --- | --- |
|  |  | **By item** | **By dimension** |  |
| Independent Living | 1 | 0 (0.0) | 2 (0.6) |  |
|  | 2 | 0 (0.0) |  |  |
|  | 3 | 2 (0.6) |  |  |
|  | 4 | 0 (0.0) |  |  |
| Relationships | 5 | 23 (7.3) | 28 (8.9) | Sexual inactivity |
|  | 6 | 1 (0.3) |  |  |
|  | 7 | 4 (1.3) |  |  |
| Mental Health | 8 | 0 (0.0) | 0 (0.0) |  |
|  | 9 | 0 (0.0) |  |  |
|  | 10 | 0 (0.0) |  |  |
|  | 11 | 0 (0.0) |  |  |
| Coping | 12 | 0 (0.0) | 2 (0.6) |  |
|  | 13 | 2 (0.6) |  |  |
|  | 14 | 0 (0.0) |  |  |
| Pain | 15 | 0 (0.0) | 0 (0.0) |  |
|  | 16 | 0 (0.0) |  |  |
|  | 17 | 0 (0.0) |  |  |
| Senses | 18 | 1 (0.3) | 2 (0.6) |  |
|  | 19 | 1 (0.3) |  |  |
|  | 20 | 0 (0.0) |  |  |
| **EQ-5D-5L** | **Item** | **Non-response – no. (%)** | | **Possible reason for non-response** |
| Mobility | 1 | 0 (0.0) | |  |
| Self-care | 2 | 0 (0.0) | |  |
| Usual Activities | 3 | 0 (0.0) | |  |
| Pain/Discomfort | 4 | 0 (0.0) | |  |
| Anxiety/Depression | 5 | 0 (0.0) | |  |
| EQ-VAS | - | 1 (0.3) | |  |

**S7.2** Multiple imputation procedure

We performed multiple imputation based on multivariate normality (MI-MVN) to address missing values in the following items: the *ind_living3 (item 3), relation1 (item 5), relation2 (item 6), relation3 (item 7), coping2 (item 13), see (item 18),* and *hear (item 19)* of the AQoL-6D, as well as *EQ-VAS*. Only one auxiliary variable, *ind_living2 (item 2)*, was used in the procedure. This variable was selected due to its lack of missing values and its high correlation with most of the aforementioned items that had missing responses. Attempts to include more than one auxiliary variable were unsuccessful due to convergence issues. Variables related to respondent characteristic were not used as auxiliary variables due to weak correlations (*r* <0.3) with the missing items. We also tried multiple imputation by chained equations (MICE) but faced difficulties with perfect prediction which persist despite various adjustments to the number and type of auxiliary variables. According to Jia and Wu (7), and Nguyen et al. (8), MI-MVN was considered an acceptable method for items with missing ordinal values, especially when MICE was not possible. Moreover, we chose to impute individual items rather than the dimension scores to allow us to demonstrate the structural model of AQoL-6D by CFA. The imputed values were rounded to the nearest response levels to enable the generation of HSUVs using the AQoL-6D utility scoring algorithm. We opted to create only 5 imputed datasets (i.e., five iterations), which is the minimum number considered acceptable by the literature (9) as our dataset exhibited low rates of missing data. Evaluation of trace plots confirmed that the imputed model exhibited acceptable convergence.

**S7.3** Robustness of the validation results to different missing data techniques

| **Missing data technique** | **Complete case analysis *(Listwise deletion)*** | | **Robust full-information maximum likelihood (RFIML)** | | **Mean imputation, then ML-SB** | | | **Multiple imputation then ML-SB** | |
| --- | --- | --- | --- | --- | --- | --- | --- | --- | --- |
| Method of imputation | NR | | No | | Mean imputation | | | Multiple imputation based on multivariate normality (MI-MVN) | |
| Method of estimation | Maximum likelihood with Satorra-Bentler correction (ML-SB) | | Robust full-information maximum likelihood (RFIML) | | ML-SB | | | ML-SB | |
| Number of subjects analysed (N) | 285 | | 314 | | 313^$^ | | | 314 | |
| HRQoL scores (Mean ± SD) | | | | | | | | | |
| EQ-VAS | 79 (17) | | 78 (17)* | | 78 (17)* | | | 79 (17) | |
| EQ-5D-5L utility | 0.837 (0.186) | | 0.829 (0.190) | | 0.829 (0.190) | | | 0.829 (0.190) | |
| AQoL-6D utility | 0.763 (0.196) | | 0.754 (0.201)*^†^ | | 0.754 (0.201)*^†^ | | | 0.753 (0.201)^$^ | |
| AQoL-6D global score | 76 (14) | | 76 (14)^#^ | | 75 (14)* | | | 75 (14) | |
| Independent Living | 72 (22) | | 71 (22) | | 71 (22) | | | 71 (22) | |
| Relationships | 79 (21) | | 79 (21) | | 78 (21)* | | | 78 (21) | |
| Mental Health | 76 (19) | | 76 (19) | | 76 (19) | | | 76 (19) | |
| Coping | 66 (22) | | 65 (22) | | 65 (22) | | | 65 (22) | |
| Pain | 77 (19) | | 77 (19) | | 77 (19) | | | 77 (19) | |
| Senses | 83 (11) | | 82 (11) | | 82 (11) | | | 82 (11) | |
| Ceiling and floor effects – no. (%) | | | | | | | | | |
| EQ-5D-5L utility | 97 (34.0) | 0 (0.0) | 102 (32.5) | 0 (0.0) | 102 (32.5) | | 0 (0.0) | 102 (32.5) | 0 (0.0) |
| EQ-VAS | 28 (9.8) | 0 (0.0) | 29 (9.3)* | 0 (0.0)* | 29 (9.3)* | | 0 (0.0)* | 29 (9.2) | 0 (0.0) |
| AQoL-6D utility | 21 (7.4) | 0 (0.0) | 21 (6.7)*^†^ | 0 (0.0)*^†^ | 21 (6.7)*^†^ | | 0 (0.0)*^†^ | 21 (6.7) | 0 (0.0) |
| AQoL-6D global score | 2 (0.7) | 0 (0.0) | 2 (0.7)^#^ | 0 (0.0)^#^ | 2 (0.6)* | | 0 (0.0)* | 2 (0.6) | 0 (0.0) |
| Independent Living | 38 (13.3) | 0 (0.0) | 39 (12.5) | 0 (0.0) | 39 (12.5) | | 0 (0.0) | 39 (12.5) | 0 (0.0) |
| Relationships | 68 (23.9) | 2 (0.7) | 68 (23.6)^#^ | 2 (0.7)^#^ | 75 (24.0)* | | 2 (0.0)* | 72 (22.9) | 2 (0.0) |
| Mental Health | 49 (17.2) | 0 (0.0) | 54 (17.2) | 0 (0.0) | 54 (17.2) | | 0 (0.0) | 54 (17.2) | 0 (0.0) |
| Coping | 19 (6.7) | 2 (0.7) | 19 (6.1) | 2 (0.6) | 19 (6.1) | | 2 (0.6) | 19 (6.1) | 2 (0.6) |
| Pain | 65 (22.8) | 0 (0.0) | 69 (22.0) | 0 (0.0) | 69 (22.0) | | 0 (0.0) | 69 (22.0) | 0 (0.0) |
| Senses | 33 (11.6) | 0 (0.0) | 35 (11.2) | 0 (0.0) | 35 (11.2) | | 0 (0.0) | 35 (11.2) | 0 (0.0) |
| Internal consistency – Cronbach’s alpha and MacDonald’s omega | | | | | | | | | |
| Independent Living | 0.89 | 0.90 | 0.89 | 0.90 | 0.89 | 0.90 | | 0.89 | 0.90 |
| Relationships | 0.74 | 0.77 | 0.74 | 0.77 | 0.77 | 0.89 | | 0.75 | 0.77 |
| Mental Health | 0.87 | 0.87 | 0.88 | 0.88 | 0.88 | 0.88 | | 0.88 | 0.88 |
| Coping | 0.72 | 0.72 | 0.72 | 0.72 | 0.72 | 0.72 | | 0.72 | 0.72 |
| Pain | 0.80 | 0.82 | 0.79 | 0.81 | 0.79 | 0.81 | | 0.79 | 0.81 |
| Senses | 0.36 | 0.44 | 0.38 | 0.45 | 0.37 | 0.45 | | 0.37 | 0.45 |
| Concurrent validity: comparison of EQ-5D-5L and AQoL-6D measures | | | | | | | | | |
| EQ-5D-5L HSUVs vs. AQoL-6D HSUVs: average ICC (95% CI) | 0.81 (0.65, 0.88), p<0.001 | | 0.81 (0.66, 0.88), p<0.001  [n=313] | | 0.81 (0.66, 0.88), p<0.001  [n=313] | | | 0.81 (0.65, 0.88), p<0.001  [n=314] | |
| EQ-VAS vs. AQoL-6D global score: average ICC (95% CI) | 0.70 (0.62, 0.76), p<0.001 | | 0.70 (0.62, 0.76), p<0.001  [n=285] | | 0.70 (0.62, 0.76), p<0.001  [n=312] | | | 0.70 (0.62, 0.76), p<0.001  [n=314] | |
| EQ-5D-5L HSUVs vs. AQoL-6D HSUVs: bias (95% LOA) | -0.074 (-0.352, 0.204) | | -0.075 (-0.354, 0.205)  [n=313] | | -0.075 (-0.355, 0.205)  [n=313] | | | -0.075 (-0.355, 0.205)  [n=314] | |
| EQ-VAS vs. AQoL-6D global score: bias (95% LOA) | -3 (-32, 26) | | -3 (-32, 26)  [n=285] | | -3 (-32, 26)  [n=312] | | | -3 (-33, 26)  [n=314] | |
| CFA – standardised loadings and standard errors: AQoL-6D < > dimension | | | | | | | | | |
| Independent Living | 0.82 | 0.03 | 0.84 | 0.04 | 0.84 | 0.03 | | 0.84 | 0.03 |
| Relationships | 0.89 | 0.03 | 0.88 | 0.03 | 0.87 | 0.03 | | 0.88 | 0.03 |
| Mental Health | 0.65 | 0.04 | 0.65 | 0.05 | 0.65 | 0.04 | | 0.65 | 0.04 |
| Coping | 0.91 | 0.04 | 0.93 | 0.04 | 0.93 | 0.04 | | 0.93 | 0.04 |
| Pain | 0.74 | 0.05 | 0.74 | 0.05 | 0.74 | 0.04 | | 0.74 | 0.04 |
| Senses | 0.70 | 0.08 | 0.72 | 0.09 | 0.73 | 0.08 | | 0.72 | 0.08 |
| CFA – standardised loadings and standard errors: Item < > dimension | | | | | | | | | |
| Independent Living < > item 1 | 0.83 | 0.02 | 0.83 | 0.02 | 0.83 | 0.02 | | 0.83 | 0.02 |
| Independent Living < > item 2 | 0.84 | 0.02 | 0.84 | 0.02 | 0.84 | 0.02 | | 0.84 | 0.02 |
| Independent Living < > item 3 | 0.86 | 0.02 | 0.86 | 0.02 | 0.86 | 0.02 | | 0.86 | 0.02 |
| Independent Living < > item 4 | 0.81 | 0.02 | 0.79 | 0.03 | 0.79 | 0.02 | | 0.79 | 0.02 |
| Relationships < > item 5 | 0.62 | 0.05 | 0.63 | 0.06 | 0.66 | 0.05 | | 0.64 | 0.05 |
| Relationships < > item 6 | 0.76 | 0.03 | 0.77 | 0.04 | 0.78 | 0.03 | | 0.77 | 0.03 |
| Relationships < > item 7 | 0.78 | 0.04 | 0.78 | 0.04 | 0.78 | 0.03 | | 0.77 | 0.03 |
| Mental Health < > item 8 | 0.78 | 0.04 | 0.79 | 0.04 | 0.79 | 0.03 | | 0.79 | 0.03 |
| Mental Health < > item 9 | 0.89 | 0.03 | 0.89 | 0.03 | 0.89 | 0.02 | | 0.89 | 0.02 |
| Mental Health < > item 10 | 0.81 | 0.03 | 0.81 | 0.03 | 0.81 | 0.03 | | 0.81 | 0.03 |
| Mental Health < > item 11 | 0.69 | 0.03 | 0.71 | 0.03 | 0.71 | 0.03 | | 0.71 | 0.03 |
| Coping < > item 12 | 0.80 | 0.03 | 0.80 | 0.03 | 0.79 | 0.03 | | 0.80 | 0.03 |
| Coping < > item 13 | 0.54 | 0.06 | 0.53 | 0.06 | 0.53 | 0.05 | | 0.53 | 0.05 |
| Coping < > item 14 | 0.65 | 0.04 | 0.66 | 0.05 | 0.66 | 0.04 | | 0.66 | 0.04 |
| Pain < > item 15 | 0.70 | 0.04 | 0.67 | 0.04 | 0.67 | 0.04 | | 0.67 | 0.04 |
| Pain < > item 16 | 0.75 | 0.03 | 0.74 | 0.03 | 0.74 | 0.03 | | 0.74 | 0.03 |
| Pain < > item 17 | 0.87 | 0.03 | 0.88 | 0.03 | 0.88 | 0.03 | | 0.88 | 0.03 |
| Senses < > item 18 | 0.34 | 0.08 | 0.34 | 0.08 | 0.35 | 0.07 | | 0.35 | 0.07 |
| Senses < > item 19 | 0.52 | 0.08 | 0.50 | 0.08 | 0.50 | 0.07 | | 0.50 | 0.07 |
| Senses < > item 20 | 0.45 | 0.08 | 0.50 | 0.08 | 0.50 | 0.07 | | 0.50 | 0.07 |
| CFA – goodness-of-fit indices | | | | | | | | | |
| χ^2^ (*df*), p-value | 352.86 (164), <0.001  χ^2^-SB (*df*), p-value: 283.67 (164), <0.001 | | NR | | 354.73 (164), <0.001  χ^2^-SB (*df*), p-value: 288.30 (164), <0.001 | | | 368.4 (164), <0.001  χ^2^-SB (*df*), p-value: 300.63 (164), <0.001 | |
| RMSEA (90% CI) | 0.064 (0.054, 0.073)  RMSEA-SB: 0.051 | | NR | | 0.061 (0.052, 0.070)  RMSEA-SB: 0.049 | | | 0.063 (0.054, 0.072)  RMSEA-SB: 0.052 | |
| pclose | 0.008 | | NR | | 0.020 | | | 0.007 | |
| CFI | 0.932  CFI-SB: 0.945 | | NR | | 0.938  CFI-SB: 0.950 | | | 0.934  CFI-SB: 0.945 | |
| TLI | 0.921  TLI-SB: 0.937 | | NR | | 0.942  TLI-SB: 0.942 | | | 0.923  TLI-SB: 0.936 | |
| SRMR | 0.058 | | Not reported because of missing values | | 0.058 | | | 0.058 | |
| CD | 0.931 | | 0.937 | | 0.937 | | | 0.938 | |

^#^n=285

*n=313

^†^One respondent had ≥2 non-response items, which rendered mean imputation (applied by the AQoL-6D utility scoring algorithm) undoable, therefore only 313 HSUVs were available.

^$^HSUVs were generated based on rounded multiply imputed values rather than mean-imputed values.

CD = coefficient of determination; CI = confidence interval; ICC = intraclass correlation coefficient; LOA = limits of agreement

**S7.4** Comparison of the validation results: base-case (n=285), sample that excluded proxy-reports (n=205), and sample that excluded respondents that required assistance when completing the survey (n=226)

| **Sample** | **Base-case** | | **Excluded proxy-reports (n=80)** | | **Excluded respondents that required assistance (n=59)** | | |
| --- | --- | --- | --- | --- | --- | --- | --- |
| Number of subjects analysed (N) | 285 | | 205 | | 226 | | |
| HRQoL scores (Mean ± SD) | | | | | | | |
| EQ-VAS | 79 (17) | | 81 (16) | | 80 (16) | | |
| EQ-5D-5L utility | 0.837 (0.186) | | 0.876 (0.164) | | 0.854 (0.178) | | |
| AQoL-6D utility | 0.763 (0.196) | | 0.794 (0.189) | | 0.773 (0.193) | | |
| AQoL-6D global score | 76 (14) | | 79 (13) | | 76 (14) | | |
| Independent Living | 72 (22) | | 78 (17) | | 73 (21) | | |
| Relationships | 79 (21) | | 82 (20) | | 79 (21) | | |
| Mental Health | 76 (19) | | 76 (20) | | 77 (19) | | |
| Coping | 66 (22) | | 69 (21) | | 67 (20) | | |
| Pain | 77 (19) | | 79 (18) | | 77 (19) | | |
| Senses | 83 (11) | | 85 (10) | | 83 (11) | | |
| Ceiling and floor effects – no. (%) | | | | | | | |
| EQ-5D-5L utility | 97 (34.0) | 0 (0.0) | 85 (41.5) | 0 (0.0) | 86 (38.1) | | 0 (0.0) |
| EQ-VAS | 28 (9.8) | 0 (0.0) | 21 (10.2) | 0 (0.0) | 21 (9.3) | | 0 (0.0) |
| AQoL-6D utility | 21 (7.4) | 0 (0.0) | 19 (9.3) | 0 (0.0) | 17 (7.5) | | 0 (0.0) |
| AQoL-6D global score | 2 (0.7) | 0 (0.0) | 2 (1.0) | 0 (0.0) | 1 (0.4) | | 0 (0.0) |
| Independent Living | 38 (13.3) | 0 (0.0) | 33 (16.1) | 0 (0.0) | 29 (12.8) | | 0 (0.0) |
| Relationships | 68 (23.9) | 2 (0.7) | 61 (29.8) | 0 (0.0) | 55 (24.3) | | 0 (0.0) |
| Mental Health | 49 (17.2) | 0 (0.0) | 38 (18.5) | 0 (0.0) | 44 (19.5) | | 0 (0.0) |
| Coping | 19 (6.7) | 2 (0.7) | 16 (7.8) | 1 (0.5) | 11 (4.9) | | 2 (0.9) |
| Pain | 65 (22.8) | 0 (0.0) | 52 (25.4) | 0 (0.0) | 53 (23.5) | | 0 (0.0) |
| Senses | 33 (11.6) | 0 (0.0) | 29 (14.2) | 0 (0.0) | 27 (12.0) | | 0 (0.0) |
| Internal consistency – Cronbach’s alpha and MacDonald’s omega | | | | | | | |
| Independent Living | 0.89 | 0.90 | 0.86 | 0.86 | 0.89 | 0.90 | |
| Relationships | 0.74 | 0.77 | 0.79 | 0.81 | 0.76 | 0.79 | |
| Mental Health | 0.87 | 0.87 | 0.88 | 0.88 | 0.87 | 0.87 | |
| Coping | 0.72 | 0.72 | 0.66 | 0.67 | 0.67 | 0.67 | |
| Pain | 0.80 | 0.82 | 0.79 | 0.81 | 0.81 | 0.83 | |
| Senses | 0.36 | 0.44 | 0.32 | Convergence not achieved | 0.40 | 0.48 | |
| EQ-5D-5L HSUVs vs. AQoL-6D HSUVs: average ICC (95% CI) | 0.81 (0.65, 0.88), p<0.001 | | 0.80 (0.55, 0.89), p<0.001 | | 0.81 (0.60, 0.89), p<0.001 | | |
| EQ-VAS vs. AQoL-6D global score: average ICC (95% CI) | 0.70 (0.62, 0.76), p<0.001 | | 0.71 (0.62, 0.78), p<0.001 | | 0.73 (0.63, 0.79), p<0.001 | | |
| EQ-5D-5L HSUVs vs. AQoL-6D HSUVs: bias (95% LOA) | -0.074 (-0.352, 0.204) | | -0.083 (-0.331, 0.165) | | -0.081 (-0.344, 0.181) | | |
| EQ-VAS vs. AQoL-6D global score: bias (95% LOA) | -3 (-32, 26) | | -2 (-29, 24) | | -4 (-31, 23) | | |
| CFA – standardised loadings and standard errors: AQoL-6D < > dimension | | | | | | | |
| Independent Living | 0.82 | 0.03 | 0.86 | 0.04 | 0.83 | 0.04 | |
| Relationships | 0.89 | 0.03 | 0.96 | 0.02 | 0.92 | 0.03 | |
| Mental Health | 0.65 | 0.04 | 0.73 | 0.04 | 0.66 | 0.05 | |
| Coping | 0.91 | 0.04 | 0.87 | 0.05 | 0.98 | 0.04 | |
| Pain | 0.74 | 0.05 | 0.76 | 0.05 | 0.07 | 0.05 | |
| Senses | 0.70 | 0.08 | 0.67 | 0.11 | 0.66 | 0.09 | |
| CFA – standardised loadings and standard errors: Item < > dimension | | | | | | | |
| Independent Living < > item 1 | 0.83 | 0.02 | 0.78 | 0.03 | 0.83 | 0.02 | |
| Independent Living < > item 2 | 0.84 | 0.02 | 0.82 | 0.03 | 0.83 | 0.02 | |
| Independent Living < > item 3 | 0.86 | 0.02 | 0.81 | 0.03 | 0.85 | 0.03 | |
| Independent Living < > item 4 | 0.81 | 0.02 | 0.74 | 0.04 | 0.81 | 0.03 | |
| Relationships < > item 5 | 0.62 | 0.05 | 0.70 | 0.05 | 0.68 | 0.05 | |
| Relationships < > item 6 | 0.76 | 0.03 | 0.77 | 0.04 | 0.75 | 0.04 | |
| Relationships < > item 7 | 0.78 | 0.04 | 0.83 | 0.03 | 0.78 | 0.04 | |
| Mental Health < > item 8 | 0.78 | 0.04 | 0.82 | 0.04 | 0.79 | 0.04 | |
| Mental Health < > item 9 | 0.89 | 0.03 | 0.91 | 0.02 | 0.88 | 0.03 | |
| Mental Health < > item 10 | 0.81 | 0.03 | 0.82 | 0.03 | 0.80 | 0.04 | |
| Mental Health < > item 11 | 0.69 | 0.03 | 0.70 | 0.03 | 0.71 | 0.03 | |
| Coping < > item 12 | 0.80 | 0.03 | 0.77 | 0.04 | 0.79 | 0.03 | |
| Coping < > item 13 | 0.54 | 0.06 | 0.45 | 0.07 | 0.45 | 0.06 | |
| Coping < > item 14 | 0.65 | 0.04 | 0.60 | 0.05 | 0.61 | 0.05 | |
| Pain < > item 15 | 0.70 | 0.04 | 0.71 | 0.04 | 0.69 | 0.04 | |
| Pain < > item 16 | 0.75 | 0.03 | 0.74 | 0.04 | 0.78 | 0.04 | |
| Pain < > item 17 | 0.87 | 0.03 | 0.86 | 0.03 | 0.88 | 0.03 | |
| Senses < > item 18 | 0.34 | 0.08 | 0.23 | 0.09 | 0.33 | 0.09 | |
| Senses < > item 19 | 0.52 | 0.08 | 0.52 | 0.11 | 0.55 | 0.08 | |
| Senses < > item 20 | 0.45 | 0.08 | 0.44 | 0.13 | 0.50 | 0.09 | |
| CFA – goodness-of-fit indices | | | | | | | |
| χ^2^ (*df*), p-value | 352.86 (164), <0.001  χ^2^-SB (*df*), p-value: 283.67 (164), <0.001 | | 353.12 (164), <0.001  χ^2^-SB (*df*), p-value: 286.91 (164), <0.001 | | 326.41 (164), <0.001  χ^2^-SB (*df*), p-value: 262.45 (164), <0.001 | | |
| RMSEA (90% CI) | 0.064 (0.054, 0.073)  RMSEA-SB: 0.051 | | 0.075 (0.064, 0.086)  0.061 | | 0.066 (0.056, 0.077)  RMSEA-SB: 0.052 | | |
| pclose | 0.008 | | <0.001 | | 0.006 | | |
| CFI | 0.932  CFI-SB: 0.945 | | 0.907  CFI-SB: 0.923 | | 0.928  CFI-SB: 0.917 | | |
| TLI | 0.921  TLI-SB: 0.937 | | 0.892  TLI-SB: 0.911 | | 0.917  TLI-SB: 0.935 | | |
| SRMR | 0.058 | | 0.058 | | 0.059 | | |
| CD | 0.931 | | 0.955 | | 0.973 | | |

CD = coefficient of determination; CI = confidence interval; ICC = intraclass correlation coefficient; LOA = limits of agreement; NR = not relevant (only size of residuals fit statistic is valid)

**References**

1. Centre for Health Economics, Monash University. Comparion of Eight Multi-Attribute Utility Instruments [Internet]. [cited 2023 Oct 7]. Available from: http://www.aqol.com.au/documents/Comparison_of_8_utility_instruments.pdf

2. Hawthorne G, Osborne R. Population norms and meaningful differences for the Assessment of Quality of Life (AQoL) measure. Australian and New Zealand Journal of Public Health. 2005;29(2):136–42.

3. Henry EB, Barry LE, Hobbins AP, McClure NS, O’Neill C. Estimation of an Instrument-Defined Minimally Important Difference in EQ-5D-5L Index Scores Based on Scoring Algorithms Derived Using the EQ-VT Version 2 Valuation Protocols. Value in Health. 2020 Jul 1;23(7):936–44.

4. Walters SJ, Brazier JE. Comparison of the minimally important difference for two health state utility measures: EQ-5D and SF-6D. Qual Life Res. 2005 Aug;14(6):1523–32.

5. Simon, M. K., White, J. Survey/Interview Validation Rubric for Expert Panel (VREP) [Internet]. Available from: http://dissertationrecipes.com/wpcontent/uploads/2011/04/Expert-Validation-v3.pdf

6. Allen J, Inder KJ, Lewin TJ, Attia JR, Kelly BJ. Construct validity of the Assessment of Quality of Life - 6D (AQoL-6D) in community samples. Health and Quality of Life Outcomes. 2013 Apr 17;11(1):61.

7. Jia F, Wu W. Evaluating methods for handling missing ordinal data in structural equation modeling. Behav Res. 2019 Oct 1;51(5):2337–55.

8. Nguyen CD, Carlin JB, Lee KJ. Practical strategies for handling breakdown of multiple imputation procedures. Emerging Themes in Epidemiology. 2021 Apr 1;18(1):5.

9. ULCA Advanced Research Computing - Statistical Methods and Data Analytics [Internet]. [cited 2023 Oct 16]. Multiple Imputation in Stata. Available from: https://stats.oarc.ucla.edu/stata/seminars/mi_in_stata_pt1_new/
